# A Single-Cell Atlas of Pan-Cancer Liver Metastasis Reveals Dynamic Cellular Programs Driving Metastatic Progression and Immune Modulation

**DOI:** 10.34133/research.1208

**Published:** 2026-03-24

**Authors:** Xinyu Tong, Haoyu Chao, Chenlu Zhang, Zhuojin Li, Quan Han, Lishan Wang, Nuo Wu, Ruidong Chen, Jian Gao, Shu Zhang, Lei Xu, Ming Chen, Hui Zhao, Lei Wang, Dijun Chen

**Affiliations:** ^1^ Department of Gastroenterology, Nanjing Drum Tower Hospital, The Affiliated Hospital of Nanjing University Medical School, Nanjing, China.; ^2^ State Key Laboratory of Pharmaceutical Biotechnology, School of Life Sciences, Nanjing University, Nanjing, China.; ^3^ Department of Bioinformatics, College of Life Sciences, Zhejiang University, Hangzhou, China.; ^4^ Department of Laboratory Diagnostics, The First Affiliated Hospital of Harbin Medical University, Harbin, China.; ^5^ Chemistry and Biomedicine Innovation Center, Nanjing University, Nanjing, China.

## Abstract

Liver metastasis remains a major challenge in cancer treatment, yet its cellular and molecular landscape remains poorly defined at the pan-cancer level. Here, we construct a single-cell transcriptomic atlas of liver metastases across multiple cancer types by analyzing 100 single-cell RNA sequencing samples, profiling over 460,000 cells, and identifying 121 distinct cellular subtypes. We define 4 representative cellular programs (CPs) associated with liver metastasis, revealing how cellular composition and intercellular interactions within the tumor microenvironment drive metastatic progression and immune modulation. These CPs recapitulate a dynamic transition from immunoactive states, marked by natural-killer-cell-mediated immune surveillance and macrophage-driven angiogenesis, to immunosuppressive environments dominated by regulatory T cell infiltration and immune exclusion. The shift is marked by progressive alterations in immune infiltration, stromal remodeling, and tumor-intrinsic adaptations, elucidating key mechanisms of immune evasion and metastatic niche formation. Our study provides a high-resolution framework for understanding the heterogeneity and evolution of liver metastasis and highlights the potential of CP-based stratification to inform therapeutic strategies targeting the metastatic tumor microenvironment.

## Introduction

Cancer metastasis remains the leading cause of cancer-related mortality, yet the mechanisms governing this complex process are not fully understood. Metastasis involves the dissemination of cancer cells from the primary tumor (PT) to distant organs, where they establish secondary tumor lesions. This multistep process includes tumor cell migration, survival in the circulation, extravasation into distant tissues, and the formation of a metastatic niche that supports tumor growth [1]. While substantial progress has been made in understanding PT biology, the metastatic process remains poorly characterized, particularly in terms of the dynamic interactions between metastatic cells and the tumor microenvironment (TME) [2]. Given the complexity of metastasis, uncovering the molecular and cellular programs (CPs) that govern this process is essential for developing effective therapeutic strategies [3].

A major breakthrough in metastasis research has been the recognition of epithelial-to-mesenchymal transition (EMT) as a key mechanism enabling cancer cells to acquire migratory and invasive properties [4]. However, metastasis extends beyond EMT alone. Cancer cells exhibit remarkable plasticity and can revert via mesenchymal-to-epithelial transition, which is critical for colonization at distant sites [5,6]. In addition, the TME, including immune cells, fibroblasts, endothelial cells, and extracellular matrix components, plays a pivotal role in metastasis by shaping tumor cell behavior and facilitating immune evasion [7]. Tumor-associated macrophages, myeloid-derived suppressor cells, and regulatory T cells (Tregs) contribute to metastatic progression by secreting cytokines and growth factors [8–10], creating an immunosuppressive niche that supports tumor survival and growth [11,12].

Despite these advances, key aspects of metastasis remain unclear. The interplay between metastatic cells and their microenvironment, as well as the specific CPs that dictate metastatic progression, requires further exploration. Notably, organ-specific metastasis presents an additional layer of complexity [12,13]. Certain cancers preferentially metastasize to specific organs, such as breast cancer to bone [14] and lung cancer to the brain [15], highlighting the role of distinct microenvironments in shaping metastatic behavior. Liver metastasis frequently occurs in multiple cancer types [16], yet its cellular and molecular landscape remains poorly understood at the systemic level.

The advent of single-cell technologies has provided unprecedented insights into tumor heterogeneity and metastatic dynamics. Single-cell RNA sequencing (scRNA-seq) enables transcriptomic profiling at the single-cell level, allowing for the identification of distinct tumor cell populations and their interactions within both primary and metastatic sites [17,18]. In addition, spatial transcriptomics preserves tissue architecture while mapping gene expression, offering valuable insights into how metastatic cells interact with their surrounding microenvironment [19–23]. Collectively, these approaches provide a high-resolution view of tumor evolution, uncovering rare cell populations and signaling pathways that contribute to metastasis.

In this study, we construct a comprehensive single-cell atlas of liver metastases across multiple cancer types, defining the dynamic CPs that drive metastatic progression and immune modulation. By integrating scRNA-seq data, we aim to map the cellular heterogeneity of liver metastases, characterize key immune and stromal interactions shaping the metastatic TME, and uncover conserved mechanisms that promote tumor progression. This work provides comprehensive insights into the heterogeneity and evolution of liver metastasis, offering novel therapeutic opportunities to target the metastatic microenvironment and improve clinical outcomes.

## Results

### A pan-cancer single-cell transcriptomic atlas of liver metastasis

**Fig. 1.**
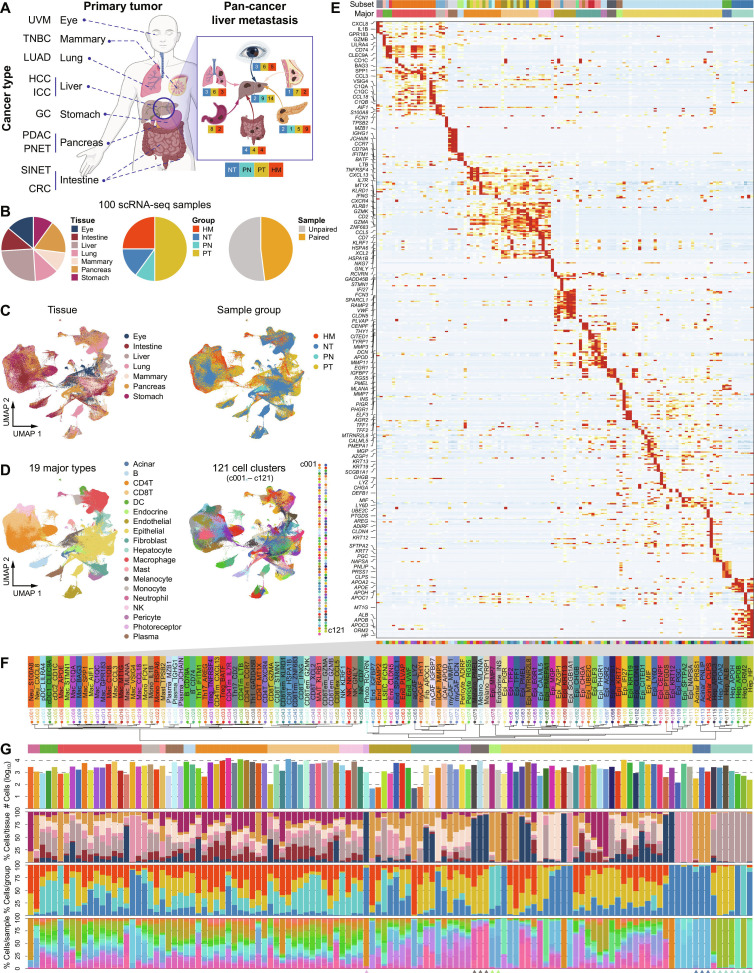
The single-cell transcriptomic landscape of pan-cancer liver metastasis. (A) The cancer types included in this pan-cancer study, color-coded by sample group. A total of 100 single-cell RNA sequencing (scRNA-seq) samples were analyzed. TNBC, triple-negative breast cancer; LUAD, lung adenocarcinoma; ICC, intrahepatic cholangiocarcinoma; PNET, pancreatic neuroendocrine tumor; SINET, small intestinal neuroendocrine tumor. (B) Pie charts showing the distribution of samples (*n* = 100) based on organ origin, health/cancer status, and the presence of matched paired samples. (C) Uniform Manifold Approximation and Projection (UMAP) plots displaying all cells from all samples, providing an overview of cohort composition. (D) UMAP plots showing major cell types and subtypes across all samples. (E) Heatmap showing expression of representative marker genes used to define annotated cell types; colors represent log-normalized expression levels. (F) Magnified view highlighting the detailed cell names presented in (E) and (G). The annotation bar shown below indicates the classification of the 121 cell clusters into major cell types [colored as shown in (D)]. (G) The top histogram presents the number of cells per cluster, normalized using log_10_. The bottom histogram illustrates the distribution of tissue origin, disease status (healthy or cancer), and individual samples within each cluster. At the bottom of the panel, clusters that are unique to a particular organ are marked.

To build a comprehensive single-cell transcriptomic landscape of pan-cancer liver metastasis, we collected scRNA-seq data from 10 different cancer types, including the 6 major PT sites of liver metastasis: eye, breast, lung, stomach, pancreas, and gastrointestinal tract. In addition to metastatic tumors (hepatic metastases [HMs]), we incorporated nonneoplastic and/or healthy tissue samples (NT/PN), with nearly half of the dataset consisting of matched tissues from the same individual. This design enabled a precise comparison of cellular composition and transcriptional programs across different conditions. Our database includes 100 scRNA-seq samples compiled from 75 individuals across 16 independent studies [24–39], representing a broad spectrum of metastatic and PT contexts (Fig. 1A and B and Table S1).

Following rigorous quality control, we identified a total of 460,337 high-quality cells for subsequent integration and analysis. To ensure data consistency across samples, we applied batch-effect correction, enabling seamless integration of datasets from different cancer types and tissue sources (Fig. 1C and Fig. S1A). Using canonical marker genes, we annotated 19 major cell types and further classified them into 34 distinct subsets with higher resolution, which were consistently identified across diverse cancer types and organ sites (Fig. S1A to C). These major cell types encompassed epithelial cells, immune cells, stromal cells, and other key components of the TME (Fig. 1D). Of note, photoreceptor cells were exclusively found in uveal melanoma (UVM), while hepatocytes were predominantly identified in liver samples, confirming the accuracy of our annotation process and the effective integration of scRNA-seq data across different cancer types and tissue sources (Fig. 1F and G and Fig. S1D). Furthermore, we systematically cross-referenced our annotations with high-quality published single-cell and spatial atlases, including the Human Cell Atlas, the TISCH (Tumor Immune Single-Cell Hub) database, and pan-cancer scRNA-seq studies [40–45]. This ensured consistent annotation of major immune, stromal, and epithelial populations across multiple cancer types (Fig. 1E and Fig. S1B and C).

To further dissect cellular heterogeneity, we subdivided the major cell types into 121 distinct cellular subtypes with cell numbers ranging from 47 to 15,640, each characterized by unique gene expression profiles (Fig. 1D to G). These clusters likely represent specialized functional states, lineage differentiation trajectories, or adaptations to the metastatic liver niche. Further hierarchical clustering of cell clusters based on pseudo-bulk expression analysis provided insights into their transcriptional relationships (Fig. 1F). In addition, we analyzed the distribution of cell proportions for each cluster across different sample groups, organ sites, and metastatic origins, revealing distinct patterns of cellular composition associated with specific tumor types and metastatic environments (Fig. 1G and Fig. S1D). For instance, we observed that certain clusters, such as those derived from immune cells, including CD4^+^ memory T (CD4Tm) cells (CD4Tm_CXCL13 and CD4Tm_LTB), T helper 1 (Th1) cells (Th1_GADD45B and Th1_IFITM1), and Tregs (Treg_TNFRSF4 and Treg_BATF), exhibited higher proportions in liver metastases originating from gastrointestinal, eye, breast, and lung cancers, suggesting a unique immune microenvironment in these metastases. Conversely, clusters related to stromal cells, including epithelial cells (Epi_TFF1, Epi_TFF2, and Epi_CHGB), antigen-presenting cancer-associated fibroblasts (apCAF_LYZ), and endothelial cells (End_IGFBP7), were more prominent in liver metastases from pancreatic cancer, indicating a stronger involvement of tissue remodeling and angiogenesis. These findings highlight the complexity of liver metastasis and underscore the importance of organ-specific and cancer-type-specific CPs that drive metastatic progression.

Collectively, this refined single-cell transcriptional landscape (hereafter referred to as the reference atlas) offers a high-resolution framework for understanding the cellular and molecular characteristics of liver metastases at the single-cell level.

### Identification of liver metastasis-associated CPs across cancer types

**Fig. 2.**
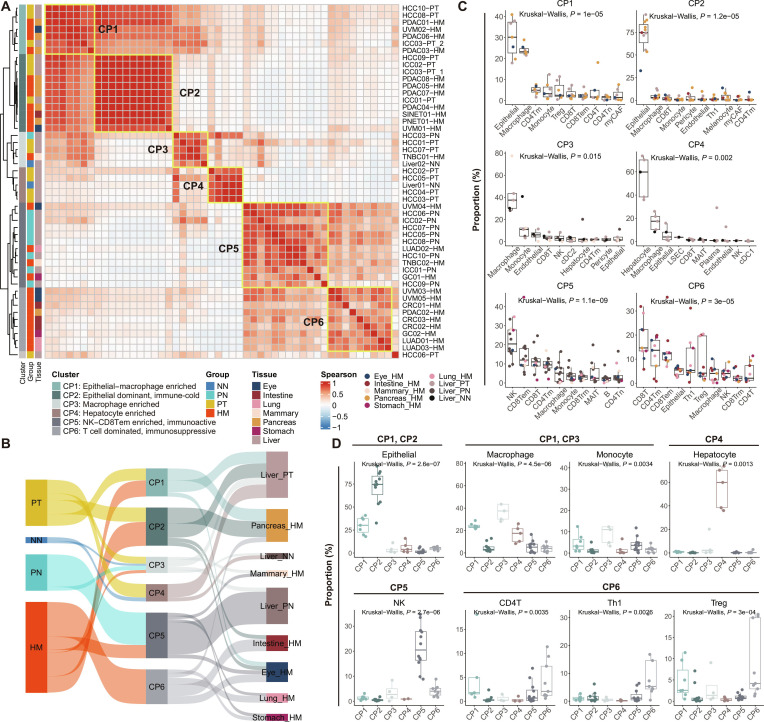
Liver-metastasis-associated cellular programs (CPs) across cancers. (A) Heatmap showing unsupervised hierarchical clustering of liver-related samples based on sample-level cellular infiltration profiles across major cell types. Pairwise sample similarity was calculated using Spearman correlation. Six recurrent CPs (CP1 to CP6) were identified and annotated with descriptive subtitles according to dominant cellular compositions: CP1, epithelial–macrophage-enriched; CP2, epithelial-dominant immune-cold; CP3, macrophage-enriched; CP4, hepatocyte-enriched; CP5, natural killer (NK) cell–CD8^+^ effector memory T (CD8Tem) cell-enriched immunoactive; CP6, T-cell-dominated immunosuppressive. Color intensity represents the Spearman correlation coefficient between samples. (B) Sankey diagram illustrating the sample composition of CPs based on health/cancer status and cancer types, highlighting how sample composition varies across CPs. (C) Box plots showing the top 10 cell types with the highest infiltration rates across each CP. CD8T, CD8^+^ T cell. The *P* values in (C) and (D) are calculated with Kruskal–Wallis test. (D) Box plots presenting the infiltration rates of key cell types across different CPs.

The preceding analysis revealed that specific cell clusters were significantly enriched in liver metastasis samples (Fig. 1G). To systematically characterize recurrent microenvironmental patterns associated with liver metastasis, we performed unsupervised clustering of liver-related samples, including liver metastases, PTs, and adjacent normal liver tissues, based on their sample-level cellular infiltration profiles. For each sample, cellular infiltration rates were quantified across major immune, stromal, and parenchymal cell types, thereby generating a compositional representation of the TME. Pairwise sample similarity was calculated using Spearman correlation, and unsupervised hierarchical clustering was applied to the resulting correlation matrix. This data-driven approach grouped samples with similar cellular compositions into 6 reproducible clusters, hereafter referred to as CPs (CP1 to CP6) (Fig. 2A). These CPs were identified on the basis of their specific cellular compositions, revealing distinct patterns of immune cell infiltration, stromal cell activity, and tumor cell abundance that distinguish liver metastasis from PTs, independent of cancer type, tissue origin, and clinical annotation. Each program likely reflects different stages of metastatic progression, immune evasion mechanisms, and adaptations to the liver microenvironment. Notably, these CPs are defined by transcriptional features and functional states shared across cancer types, rather than their anatomical origin, offering valuable insights into the factors driving liver metastasis across various cancer types. Specifically, we found that both primary liver tumors and pancreatic cancer liver metastases were predominantly associated with CP1 and CP2, while eye-derived metastatic samples exhibited a more dispersed distribution pattern across 4 programs (CP1, CP2, CP5, and CP6; Fig. 2B). Notably, CP5 and CP6 were highly enriched in liver metastases from various PTs. CP6 was exclusively composed of liver metastases, except for those derived from breast cancer (Fig. 2B), suggesting a distinct CP potentially linked to more aggressive or clinically lethal metastatic progression. In contrast, CP5 included both adjacent normal liver samples and liver metastases primarily from breast, lung, and stomach cancers, implying that CP5 may be associated with a more favorable clinical prognosis or a less aggressive metastatic phenotype.

The above observations were consistent with the relative contributions of cell types in each CP (Fig. 2C and D). For example, CP5, which is predominantly composed of liver metastases associated with a more favorable prognosis, exhibited significant infiltration of natural killer (NK) cells and CD8^+^ effector memory T (CD8Tem) cells (Fig. 2C and D), both of which are key players in antitumor immunity [46,47]. The presence of these immune cells suggests a more active immune surveillance state, potentially contributing to better disease outcomes [48,49]. In contrast, CP6 was characterized by a dominant infiltration of multiple T cell subtypes, including CD8^+^ T cells, CD4Tm cells, Th1 cells, and Tregs (Fig. 2C and D). The enrichment of Th1 cells indicates a proinflammatory response, whereas the presence of Tregs suggests a concurrent immunosuppressive environment [50,51]. This dual landscape may reflect an immune-evasive TME, where immune activation coexists with mechanisms of immune suppression, potentially driving more aggressive metastatic progression [52–54].

Of note, CP3 and CP4, which were primarily associated with primary cancers and adjacent normal liver tissues (Fig. 2A and B), exhibited distinct cellular compositions. CP3 was characterized by a dominant infiltration of macrophages (Fig. 2C and D), consistent with their established role in shaping the TME through both pro- and antitumor functions [55]. The enrichment of macrophages in CP3 suggests their potential involvement in tumor-associated inflammation, immune modulation, and stromal remodeling, which may influence tumor progression and therapeutic responses [56,57]. In contrast, CP4 was enriched in hepatocytes (Fig. 2C and D), aligning with its association with low-grade tumors or nonneoplastic liver tissues (Table S1), further validating the robustness of our classification.

In addition, both CP1 and CP2 exhibited significant epithelial cell infiltration (Fig. 2C and D). However, CP2 lacked infiltration of immune cells, which may reflect a “cold tumor” phenotype, known for its poor immune infiltration and resistance to immunotherapy [58,59]. In contrast, CP1, while sharing these features, also displayed increased macrophage infiltration, suggesting an additional immune component that may contribute to tumor progression, immune evasion, or potential therapeutic vulnerabilities [60–62]. This dual presence of epithelial cells and macrophage cells in CP1 may reflect a transition toward a more immune-enriched TME, possibly indicating a shift from “cold” to “hot” tumor features that could influence both immune evasion and treatment responsiveness.

In summary, the above findings highlight the diversity of CPs present in liver metastases and suggest distinct microenvironmental adaptations across different metastatic subtypes. In the following sections, we will elaborate on key cell subtypes within these CPs, focusing on their roles in immune modulation, metastatic progression, and potential therapeutic targets associated with liver metastases.

### Copy number variation analysis provides a genomic framework for epithelial cell heterogeneity in liver metastasis

To establish a genomic framework supporting subsequent analyses of CPs, we first focused on epithelial cells, which are predominantly enriched in CP1 and CP2, by examining copy number variations (CNVs) across different tumor samples. We therefore used inferCNV [63] to detect CNV landscapes in individual tumor samples and performed clustering analysis in Uniform Manifold Approximation and Projection (UMAP) embedding space based on estimated CNV scores, allowing us to compare the genomic variation patterns among different tumors. This analysis grouped 105,460 epithelial cells into 66 CNV clusters, each displaying a unique genomic alteration profile (Fig. S2A). Overall, each tumor exhibited distinct CNV patterns, highlighting the heterogeneity of genomic alterations across different metastatic tumors and PTs (Fig. S2B). The CNV profiles demonstrated a cancer-type-specific pattern and varied significantly among adjacent normal tissues, PTs, and liver metastases, suggesting that the genomic landscape of epithelial cells evolves throughout tumor progression (Fig. S2B). Notably, these CNV clusters were largely consistent with epithelial cell subtypes, as shown by subtype annotation of CNV clusters and their correspondence across datasets (Figs. S2C and S3A), suggesting a strong link between genomic alterations and cellular phenotypes. This finding implies that specific CNV patterns may underlie functional differences among epithelial cells, potentially influencing tumor progression, metastatic potential, and treatment responses.

Interestingly, we found that the CNV patterns of adjacent normal samples tended to cluster toward the center of the UMAP space, whereas those of PTs and liver metastases were more dispersed (Fig. S2B). To further investigate these differences, we compared the genomic variation patterns between paired samples from the same patients. This allowed us to assess the degree of genomic divergence both between PTs and their corresponding liver metastases and between PTs and adjacent normal tissues (Fig. S2D). As expected, PTs exhibited significantly higher CNV scores than adjacent normal tissues, indicating a higher level of genomic instability and alterations in PTs compared to the relatively stable normal tissue. Notably, liver metastases exhibited even lower CNV scores compared to PTs derived from eye, intestine, and stomach cancers, suggesting a possible selective evolutionary process in metastasis. However, a reversed trend was observed in pancreatic cancer, where liver metastases showed significantly higher CNV scores compared to their PTs [35] (Fig. S2D). This is consistent with the observation that liver metastasis in pancreatic cancer may involve a more complex or distinct genomic evolution compared to other cancer types [64,65]. These observations underscore the dynamic nature of tumor evolution, where liver metastases may experience further genetic diversification and selection pressures compared to their primary counterparts, potentially influencing metastatic behavior and therapeutic responses.

To further quantify the genomic heterogeneity of cancer cells, we calculated entropy across CNV-defined clusters within each sample and summarized the results across different CPs. Here, entropy is used as an information-theoretic measure to quantify the diversity of cancer cells distributed across distinct CNV-defined clusters, with higher entropy indicating a more heterogeneous and genomically diverse CNV landscape within a sample. Entropy thus serves as a quantitative measure of genomic instability and complexity, providing insights into the degree of heterogeneity associated with metastatic progression. We observed that the entropy scores for CP5 and CP6 were generally higher compared to CP1 and CP2 based on CNV-defined epithelial cell clusters (Fig. S2E). This pattern supports the notion that CP5 and CP6 represent more heterogeneous and genomically unstable phases of metastasis, marked by greater genomic diversity and potential ongoing evolutionary processes within metastatic sites. In contrast, CP1 and CP2 appear to reflect more differentiated and stable states, characterized by lower genomic variability and a more fixed cellular identity.

To identify specific genomic alterations that may drive the metastatic process or confer advantages to tumor cells during their colonization of the liver microenvironment, we compared the differences in CNV profiles between liver metastasis samples and the corresponding PTs. In total, 181 genes exhibiting significant alterations in at least one organ site were identified, which were subsequently categorized into 4 groups (K1 to K4) based on the differential CNV scores (Fig. S2F). Further functional enrichment analysis was performed separately for each gene group (K1 to K4), revealing distinct pathway enrichment patterns associated with their differential CNV alteration profiles (Fig. S2G). Notably, interferon signaling, antigen processing, and presentation pathways were highly enriched in the groups of genes (K2 and K3), showing reduced CNV alteration in pancreatic cancer liver metastasis. This suggests that liver metastasis in pancreatic cancer may involve a specific down-regulation of immune response pathways, potentially contributing to immune evasion and promoting metastasis in the liver microenvironment. In pancreatic cancer samples, the K4 gene set showed significantly elevated expression. Pathway enrichment analysis revealed that K4 was primarily associated with tumor cell–intrinsic regulatory pathways, including RNA surveillance and catabolic processes, epigenetic regulation, and mitochondrial translation (Fig. S2G). These features suggest enhanced transcriptional control and metabolic adaptation of cancer cells, distinguishing K4 from immune-associated gene programs. This observation aligns with the immune cell infiltration deficiency observed in CP2 (Fig. 2C), which could be related to the loss of antigen presentation genes induced by malignant epithelial cells in pancreatic cancer liver metastasis. These findings highlight potential pathways that could be targeted to mitigate metastatic progression and improve therapeutic strategies for liver metastasis. Together, these CNV-based analyses provide a genomic context for epithelial cell heterogeneity in liver metastasis, while detailed CNV cluster composition, pseudotime ordering, and pathway enrichment analyses are presented in Fig. S3.

### Trajectory analysis revealing cellular processes driving metastatic progression

**Fig. 3.**
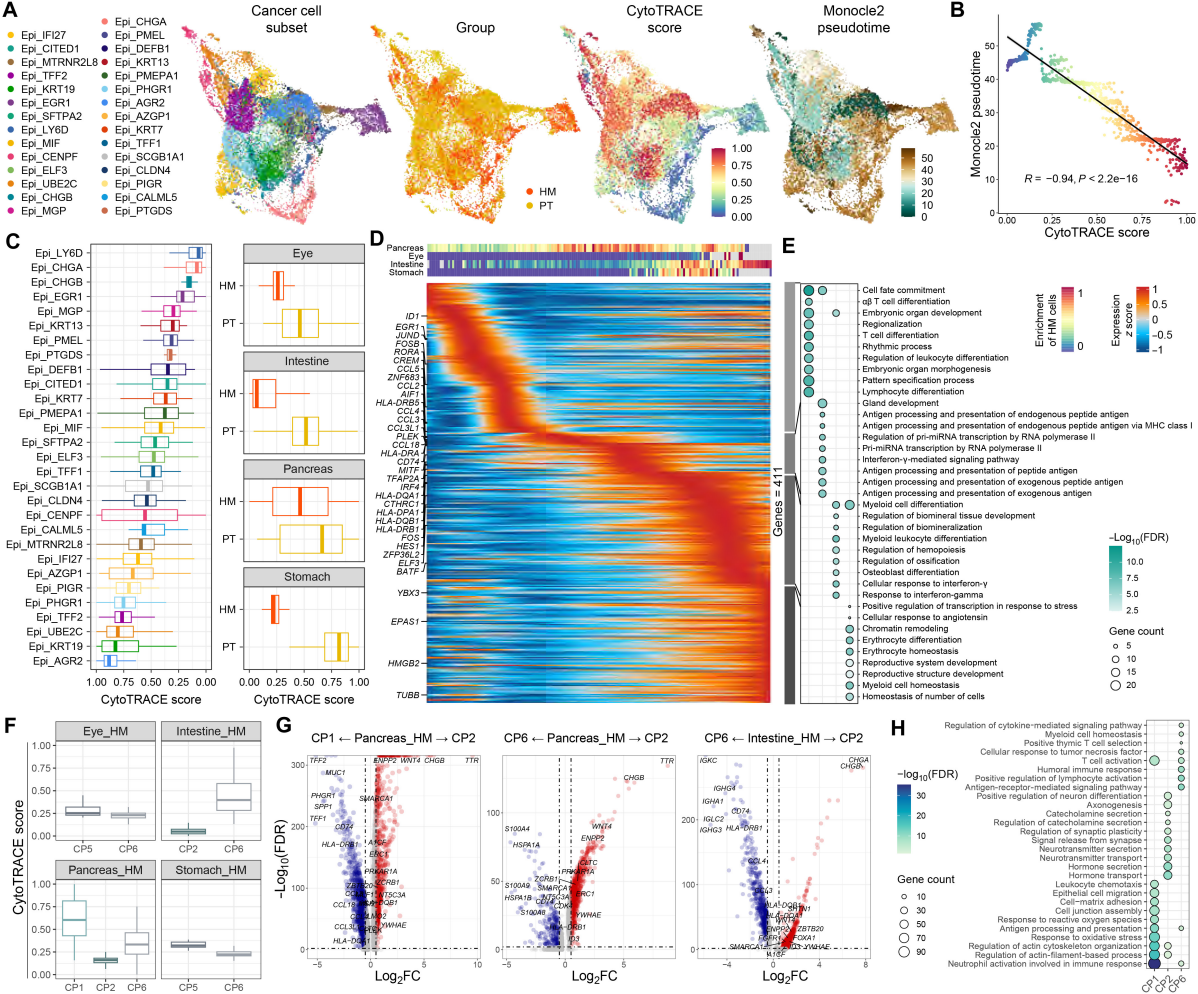
Pseudotime trajectory analysis reveals epithelial cellular processes driving metastatic progression. (A) Uniform Manifold Approximation and Projection (UMAP) plots of all epithelial cells from liver metastases and their matched primary tumors (PTs) used in CytoTRACE and Monocle2 pseudotime analysis. (B) The developmental pseudotime of epithelial cells is negatively correlated with the CytoTRACE score. (C) Box plots showing CytoTRACE scores of epithelial cell subpopulations and epithelial cells from different organ origins. (D) The top heatmap depicts the distribution of hepatic metastasis (HM) cells across different organ origins. The bottom heatmap shows the scaled expression of differentially expressed genes along the pseudotime trajectory shown in (A). (E) Dot plot displaying gene ontology analysis of the clusters identified in (D) using clusterProfiler. The size and color of the dot represent the number of enriched genes and adjusted *P* value from one-sided Fisher’s exact test, adjusted with the Benjamini–Hochberg method, respectively. MHC, major histocompatibility complex; pri-miRNA, primary microRNA; FDR, false discovery rate. (F) Box plots showing CytoTRACE scores of liver metastasis samples across different cellular programs (CPs) for each organ origin. (G) Volcano plots highlighting genes specifically expressed in CP2 compared to other CPs of liver metastasis samples. Log_2_FC, log2 fold change. (H) Dot plot displaying gene ontology analysis of CPs in (G) using clusterProfiler. The size and color of the dots represent the number of enriched genes and *P* value from one-sided Fisher’s exact test, adjusted with the Benjamini–Hochberg method, respectively.

To uncover the differentiation dynamics and potential trajectory of epithelial cell subtypes in liver metastasis, we utilized both CytoTRACE [66] and Monocle2 [67] to assess the developmental states and pseudotime trajectories of all 29 epithelial cell clusters (Fig. 3A and Fig. S3B). Both analyses yielded highly consistent results: Cells with lower CytoTRACE scores (i.e., lymphocyte antigen 6 family member D-positive [LY6D^+^] epithelial cells), indicating more differentiated states, were associated with later stages of pseudotime, while cells with higher CytoTRACE scores (anterior gradient 2-positive [AGR2^+^] epithelial cells), representing less differentiated states and corresponding to earlier stages in Monocle2 pseudotime progression (Fig. 3B). Notably, *AGR2* has been implicated in promoting cancer metastasis [68]. As expected, liver metastatic cancer cells exhibited significantly lower differentiation potential compared to PT cells, corresponding to their positioning at the later stages of the trajectory. This supports the notion that metastatic tumors may arise through progressive differentiation from PTs (Fig. 3C). These results confirm that the differentiation trajectory of epithelial cells follows a clear developmental progression, a process that may be crucial for understanding tumor evolution and metastasis.

To recapitulate the molecular programs along the pseudotime progression, we performed differential expression analysis by regressing gene expression against pseudotime to identify key gene expression changes associated with epithelial cell differentiation in liver metastasis. The differentiation trajectory of cancer cells mirrored metastatic progression, characterized by the up-regulation of genes involved in cell differentiation and transcriptional regulation (*ID1* and *ELF3*), immune regulation and inflammation (*CCL5* and *HLA-DRA*), EMT (*CTHRC1*), and metabolic adaptation and hypoxia response (*EPAS1* and *HMGB2*) (Fig. 3D). Notably, pathways related to immune modulation, such as T cell differentiation, antigen processing and presentation, and regulation of leukocyte differentiation, were enriched along the pseudotime trajectory, suggesting that immune evasion mechanisms may play a crucial role in metastatic progression (Fig. 3E). In addition, genes associated with stemness and epithelial plasticity (*HES1* and *ZFP36L2*) were dynamically regulated, suggesting that metastatic cells may undergo differentiation-related changes to enhance their adaptability in the liver microenvironment. EMT-associated genes, such as *CTHRC1* and *FOS* were up-regulated along the trajectory, supporting the notion that metastatic cells may acquire mesenchymal-like properties to facilitate invasion and dissemination.

Furthermore, we calculated the CytoTRACE scores for epithelial cells from liver metastasis samples derived from intestinal and pancreatic origins, revealing a conserved progressive transition through CPs from CP6 to CP2 (Fig. 3F). This observation suggests a dynamic differentiation trajectory in which cells transition from a less differentiated state in CP6, characterized by immune-enriched TMEs, to more differentiated and metastatic phenotypes in CP2, characterized by immune-desert microenvironments (Fig. 2C). Of note, CP1 cancer cells exhibited an even lower differentiation state than CP6 and CP2, suggesting a potential early-stage or less differentiated subpopulation within the metastatic niche (Fig. 3F). Further comparative analysis of gene expression across CPs revealed that cancer cells in CP2 exhibited reduced expression of genes involved in antigen processing and presentation compared to CP1 and CP6, a pattern conserved in both intestinal and pancreatic cancer liver metastases (Fig. 3G and H and Fig. S3C). In contrast, pathways related to neurotransmitter secretion, synaptic plasticity regulation, axon formation, and catecholamine secretion were up-regulated in CP2. These findings suggest that CP2 may represent a stage in which cancer cells evade immune surveillance by suppressing antigen presentation while simultaneously co-opting neural signaling pathways to drive tumor progression and adaptation within the metastatic niche [69,70].

### CP1-associated macrophage subtypes in immune modulation and metastatic progression

**Fig. 4.**
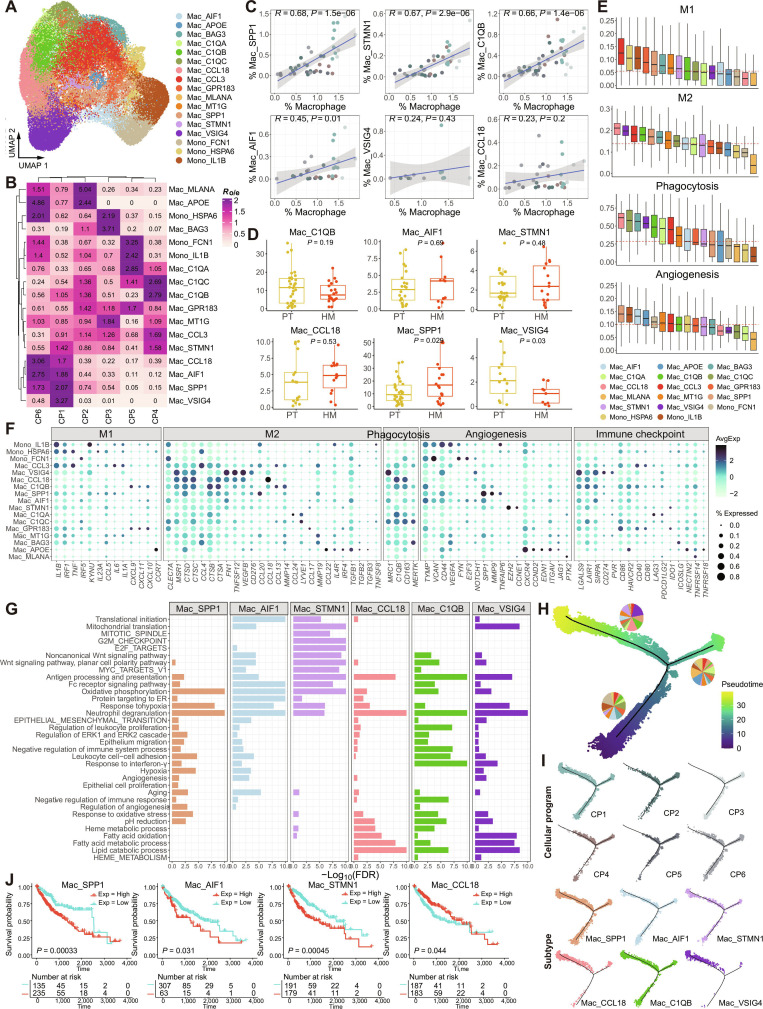
Characteristics of macrophage/monocyte cells in the tumor microenvironment (TME) of liver-related samples. (A) Uniform Manifold Approximation and Projection (UMAP) plot displaying macrophage subtypes across all samples. (B) Heatmap showing enrichment of clusters in CP1-associated cellular programs (CPs), quantified by *R*_o/e_ (see Materials and Methods). (C) Scatterplots showing correlations between the infiltration rates of macrophages in the TME and those of specific macrophage/monocyte subtypes. (D) Box plots comparing the proportions of specific macrophage/monocyte clusters in primary tumors (PTs) and liver metastases. The *P* values were calculated using the Kruskal–Wallis test. (E) Box plots showing the function signature score (see Materials and Methods) for each macrophage/monocyte subtype. The red dashed line represents the median value of all subtypes. (F) Detailed presentation of gene sets used for functional signature scoring in (E). (G) Bar plot displaying gene ontology and Hallmark analysis of macrophage/monocyte subtypes using clusterProfiler. ER, endoplasmic reticulum; ERK1, extracellular-signal-regulated kinase 1. (H) Pseudotime trajectory plot illustrating the developmental progression of macrophage subtypes, with pie charts showing the subtype composition in each branch. (I) Pseudotime trajectory plots showing the developmental progression of different CPs and subtypes. (J) Kaplan–Meier overall survival curves of The Cancer Genome Atlas-liver hepatocellular carcinoma (TCGA-LIHC) patients, stratified by high versus low abundance of CP1-associated macrophage/monocyte subtypes (mean abundance as cutoff), reflecting the liver-specific immune context of CP1-associated macrophages. Numbers at risk are shown below each curve. *P* values were calculated using a 2-sided log-rank test. The analysis included 370 patients, with a median follow-up time of 21.0 months (range, 0 to 120.7 months) and 130 observed events.

The above analysis indicates that the macrophage-associated CP1 represents an immune-enriched state within the metastatic niche, where cancer cells coexist with infiltrating macrophages in an early-stage TME. To further explore the role of macrophages in this process, we sought to investigate the distribution and functional characteristics of macrophage subtypes (*n* = 17) in liver metastases (Fig. 4A). Using ratios of observed cell numbers to random expectations (*R*_o/e_) analysis [71,72] (see Materials and Methods), we identified 6 macrophage subtypes (comprising 35.3% of the macrophage population; including Mac_C1QB, Mac_STMN1, Mac_CCL18, Mac_AIF1, Mac_SPP1, and Mac_VSIG4) that were enriched in CP1 (Fig. 4B). Notably, most of these subtypes showed a significant positive correlation with overall macrophage infiltration in the TME (Fig. 4C and Fig. S4A), suggesting their potential involvement in immune modulation during metastatic progression. Among them, the subtype of Mac_SPP1 exhibited a marked expansion in the liver metastatic microenvironment, whereas Mac_VSIG4 was significantly depleted in liver metastases (Fig. 4D).

To further explore the functions of macrophage subtypes, we calculated gene module scores [73] using known macrophage markers for M1, M2, phagocytosis, and angiogenesis [45,74–76]. Our analysis revealed that CP1-associated macrophage subtypes Mac_SPP1 and Mac_AIF1 exhibited the highest angiogenesis potential among the subtypes. Conversely, Mac_C1QB, Mac_VSIG4, and Mac_CCL18 not only demonstrated strong phagocytic activity but also showed elevated M2 polarization (tumor-promoting phenotype) scores (Fig. 4E). Notably, Mac_STMN1 specifically overexpressed *EZH2* and *CCNE1* (Fig. 4F). *EZH2* is involved in hematopoiesis [77], while *CCNE1*, a cyclin, has been implicated in various cancers [78], suggesting that Mac_STMN1 may contribute to tumor progression. In addition, gene set enrichment analysis identified both shared and distinct pathways among CP1-associated macrophage subtypes (Fig. 4G). For instance, all the 6 macrophage subtypes were significantly enriched in pathways associated with oxidative phosphorylation, hypoxia response, and antigen processing and presentation. Mac_SPP1, Mac_AIF1, and Mac_VSIG4 exhibited enrichment in pathways associated with interferon-γ response, leukocyte cell–cell adhesion and EMT. Meanwhile, Mac_CCL18, Mac_C1QB, and Mac_VSIG4 were enriched in pathways associated with heme metabolism, lipid metabolism, and fatty acid oxidation (Fig. 4G), indicating their potential roles in shaping a pro-TME by supporting metabolic adaptation and angiogenesis [64]. Together, these findings offer valuable insights into the distinct functional roles of macrophage subtypes in liver metastasis, highlighting their potential contributions to immune modulation and metastatic progression.

The divergent roles of CP1-associated macrophage subtypes were further supported by pseudotime trajectory analysis, which revealed that these subtypes spanned the entire developmental trajectory (Fig. 4H). Mac_AIF1 was enriched at early stages and appeared to give rise to Mac_SPP1 and Mac_STMN1 at later stages. In contrast, Mac_CCL18 was positioned at the terminal stage of the trajectory, suggesting its role in late-stage tumor progression (Fig. 4I). Subsequent survival analysis showed that high expression of Mac_SPP1, Mac_STMN1, and Mac_AIF1 was significantly correlated with poor prognosis in The Cancer Genome Atlas (TCGA) liver dataset (Fig. 4J). Given the liver-enriched and tissue-resident characteristics of several CP1-associated macrophage subtypes, TCGA-liver hepatocellular carcinoma (LIHC) was selected to evaluate their prognostic relevance within a hepatic immune microenvironment. In contrast, high expression of Mac_CCL18 correlated with favorable overall survival at early stages but showed a sharp decline in survival at later stages. This may be attributed to Mac_CCL18’s normal role in maintaining vascular function in the liver, which, as the disease progresses, is hijacked by cancer cells to facilitate angiogenesis and tumor metabolism, ultimately driving tumor progression.

### CP5-associated NK cells linked to favorable overall survival outcomes

**Fig. 5.**
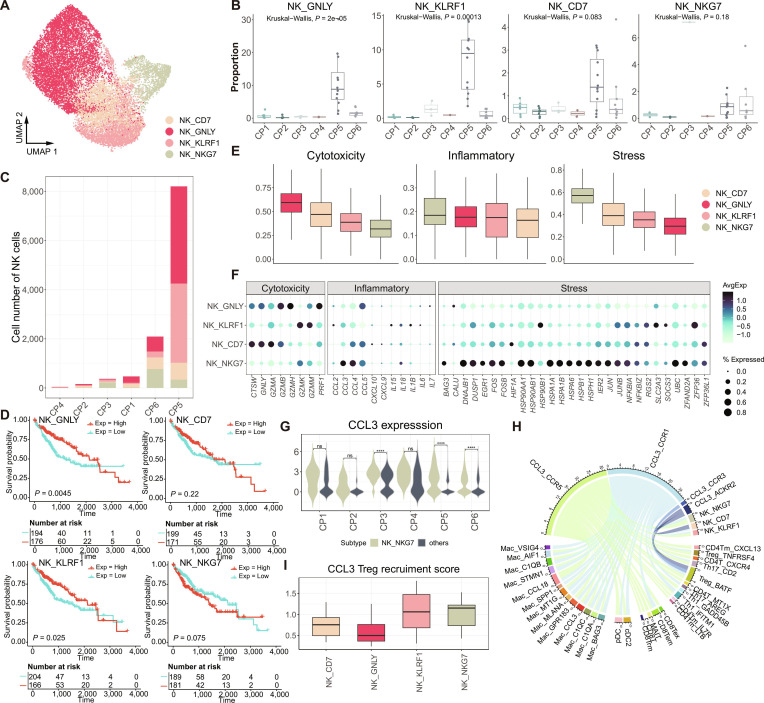
Characteristics of natural killer (NK) cells in the tumor microenvironment (TME) of liver-related samples. (A) Uniform Manifold Approximation and Projection (UMAP) plot displaying NK subtypes across all samples. (B) Box plots showing the proportion of each NK subtype in different cellular programs (CPs). The *P* values are calculated using Kruskal–Wallis test. (C) Bar plot showing the absolute cell number of NK cells in TME of each CP. (D) Kaplan–Meier overall survival curves of The Cancer Genome Atlas-liver hepatocellular carcinoma (TCGA-LIHC) patients stratified by gene set variation analysis (GSVA) scores of NK-cell-subtype-specific gene signatures (mean score as cutoff). Numbers at risk are shown below each curve. *P* values were calculated using a 2-sided log-rank test. The analysis included 370 patients, with a median follow-up of 21.0 months (range, 0 to 120.7 months) and 130 observed events. (E) Box plots showing functional module scores (cytotoxicity, inflammatory, and stress) across each NK subtype (see Materials and Methods). (F) Detailed presentation of the gene sets used in (E). (G) Violin plot showing CCL3 expression in different CPs. ns, not significant. ****P < 0.0001. (H) Chord diagram showing ligand–receptor (LR) interactions between NK subtypes and other immune cell populations, highlighting CCL3-mediated communication. (I) Box plots of the CCL3–regulatory T cell (Treg) recruitment score for each NK subtype, quantifying the potential of each NK subtype to recruit Tregs in the TME.

We next focused on the subtypes of NK cells and identified 4 distinct NK cell subtypes (Fig. 5A), all of which predominantly infiltrated CP5 (Fig. 5B and C). Survival analysis revealed that all subtypes, except for NK_NKG7, were significantly associated with a more favorable prognosis (Fig. 5D), supporting the notion that CP5 is indicative of a less aggressive metastatic phenotype and correlates with better overall survival. NK-cell-associated programs represent relatively general antitumor immune states that are less dependent on organ-specific microenvironments. Their prognostic relevance was therefore evaluated using the TCGA-LIHC cohort to maintain consistency with macrophage-associated analyses and to enable direct comparison of immune programs within a unified liver metastatic context. Note that the NK_NKG7 subtype was also the predominant NK cell subtype in CP6 (Fig. 5B). This subtype exhibited high cytotoxicity, elevated inflammatory activity, and low-stress-related functional characteristics (Fig. 5E). In addition, NK_NKG7 exhibited elevated expression of chemokines, including *CCL3*, *CCL4*, and *CCL5* (Fig. 5F), with *CCL3* expression being notably higher compared to other NK cell subtypes across most CPs (Fig. 5G). This suggests a potential role of NK_NKG7 in shaping the immune landscape of the TME, particularly through recruitment of immune cells mediated by *CCL3* and other chemokines.

To explore this further, we analyzed cell–cell communication interactions related to *CCL3*. Interestingly, we found that NK_NKG7 exhibited active communication with Tregs (Fig. 5H), and this interaction was notably stronger compared to other NK subtypes and Tregs (Fig. 5I). Since NK_NKG7 showed a trend toward poorer overall survival (Fig. 5D), it may contribute to immune tolerance within the liver metastatic TME by recruiting Tregs, potentially promoting immune evasion and leading to poorer cancer survival outcomes.

### CP6-associated CD4 T cell subtypes and their interactions in liver metastasis

**Fig. 6.**
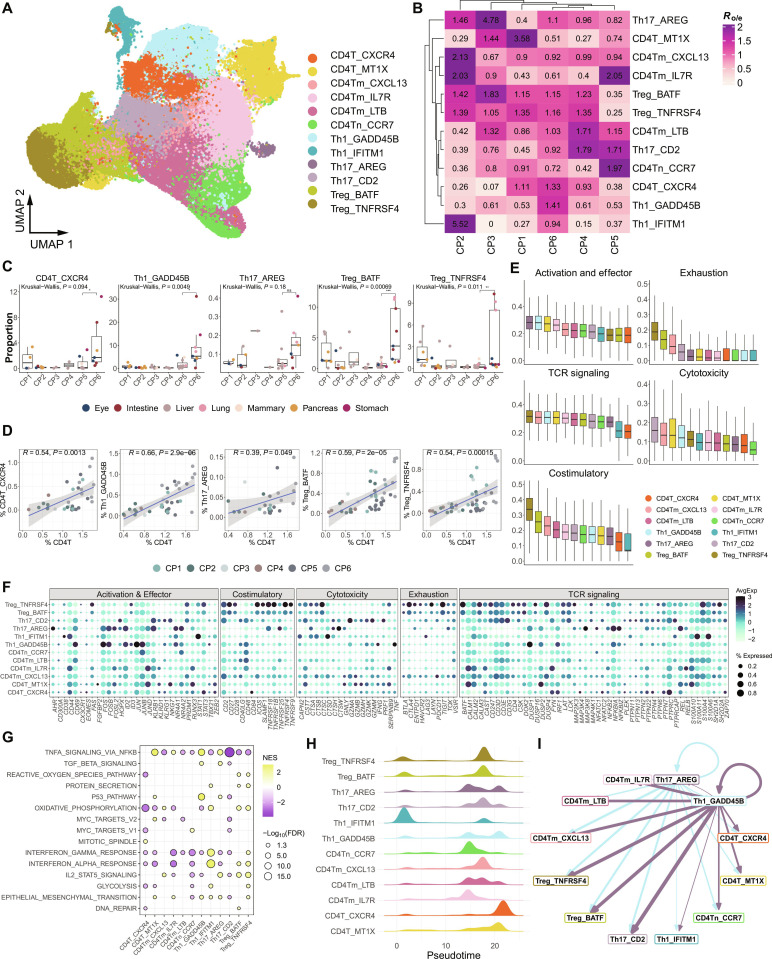
Characteristics of CD4^+^ T cells in the tumor microenvironment (TME) of liver-related samples. (A) Uniform Manifold Approximation and Projection (UMAP) plot displaying CD4^+^ T cell subtypes across all samples. (B) Heatmap showing cluster enrichment for different cellular programs (CPs; CP1 to CP6) calculated using *R*_o/e_ (see Materials and Methods). (C) Box plots comparing the proportion of certain CD4^+^ T cell subtypes in different CPs. The *P* values are calculated using Kruskal–Wallis test. *P < 0.05; **P < 0.01; ***P < 0.001. (D) Scatterplots showing correlations between the infiltration rates of CD4^+^ T cells in the TME and those of specific CD4^+^ T cell subtypes. (E) Box plots of functional module scores for each subtype, including activation, exhaustion, T cell receptor (TCR) signaling, cytotoxicity, and costimulation. (F) Detailed presentation of the gene sets used in (E). (G) Dot plot displaying Hallmark gene set enrichment analysis of CD4^+^ T cells using clusterProfiler. The color and size of the dot represent normalized enrichment score (NES) score and the adjusted *P* value from one-sided Fisher’s exact test, adjusted with the Benjamini–Hochberg method, respectively. (H) Ridgeline plot showing the developmental pseudotime trajectory of CD4^+^ T cell subtypes. (I) Chord diagram showing the cell–cell interaction strength between Th17_AREG, Th1_GADD45B, and other CD4^+^ T cell subtypes based on LR analysis.

We then investigated the CD4 T cell subtypes, which were highly infiltrated in CP6 (Fig. 2D). In total, we identified 12 distinct CD4 T cell subtypes in the reference atlas (Fig. 6A). Among them, CD4T_CXCR4, Th1_GADD45B, Th17_AREG, Treg_TNFRSF4, and Treg_BATF were the most enriched in CP6 (Fig. 6B). Notably, the 2 Treg subtypes were uniquely associated with the immune context of CP6 compared to CP5 (Fig. 6C). The infiltration levels of these 5 subtypes showed a significant positive correlation with overall CD4 T cell infiltration in the TME (Fig. 6D), indicating their key roles within CP6. Consistent with this, the Th17_AREG subtype showed elevated expression of genes related to cellular activation and effector functions, such as *FOS/FOSB*, *ID2*, *JUNB/JUND*, and *NR4A1/NR4A2* (Fig. 6E and F). Meanwhile, Th1_GADD45B was characterized by elevated cellular activation, effector function, and T cell receptor (TCR) signaling. In addition, both Treg_TNFRSF4 and Treg_BATF displayed the highest levels of exhaustion and costimulatory activity, with Treg_TNFRSF4 also exhibiting the strongest TCR signaling activity, indicating high functional activity within the CP6 TME. Accordingly, Treg and Th1_GADD45B subtypes highly expressed genes associated with pathways related to energy supply, EMT, and interferon response (Fig. 6G). Notably, the Th1_GADD45B and Treg subtypes were specifically enriched in transforming growth factor-β (TGF-β) signaling and interleukin-2 (IL-2)–signal transducers and activators of transcription 5 (STAT5) signaling pathways, respectively.

In contrast, CD4T_CXCR4 exhibited the lowest levels of activation, TCR signaling, exhaustion, cytotoxicity, and costimulatory activity among nearly all subtypes (Fig. 6E). It also showed reduced cell cycle activity and lower energy demands (Fig. 6G). Pseudotime analysis revealed that this subtype primarily emerged in the later stages of development (Fig. 6H). These findings suggest that CD4T_CXCR4 may represent a functionally impaired, low-response CD4 T cell subtype that arises in the later stages of liver metastasis within the TME. Interestingly, Th1_GADD45B and Th17_AREG displayed similar pseudotime patterns to CD4T_CXCR4 (Fig. 6H), suggesting potential overlapping or sequential roles in the immune progression within the metastatic TME.

Indeed, Th1_GADD45B and Th17_AREG exhibited strong cell–cell interactions with CD4T_CXCR4 and Treg subtypes (Fig. 6I), emphasizing their potential collaborative roles in shaping the immune landscape during liver metastasis. We speculate that the Th1_GADD45B and Th17_AREG subtypes may contribute to Treg differentiation and enhance tumor cell invasion and metastasis within the TME. These interactions likely serve as key drivers of the immunosuppressive environment in the late stages of liver metastasis.

### Cell–cell communications within CPs associated with liver metastasis

**Fig. 7.**
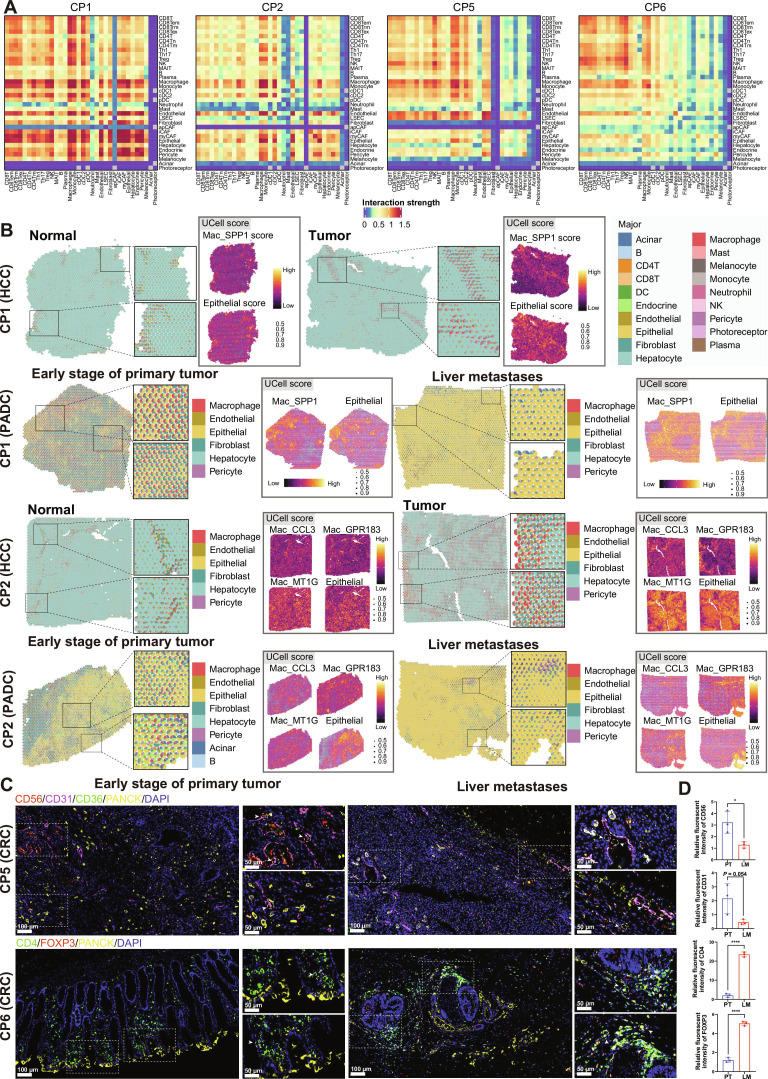
Cell–cell interaction networks in the tumor microenvironment (TME) within different cellular programs (CPs). (A) Heatmaps illustrating the cell–cell interaction patterns in representative CP1, CP2, CP5, and CP6 based on CellPhoneDB analysis. CD8Trm, CD8 tissue-resident memory T cell; CD8Tn, CD8 naïve T cell; MAIT, mucosal-associated invariant T cell; iCAF, inflammatory cancer-associated fibroblast. (B) Spatial transcriptomic maps from paired liver metastasis samples of hepatocellular carcinoma (HCC) and pancreatic ductal adenocarcinoma (PDAC) showing cell type composition inferred by single-cell-based spatial deconvolution (left) and UCell enrichment scores for CP1- and CP2-associated macrophage programs and epithelial signatures (right). Spatial data were derived from paired samples of the same patients. Color intensity and point size indicate relative enrichment score. (C) Representative immunofluorescence images showing marker expression patterns for tumor epithelial cells (PANCK), endothelial cells (CD31), NK cells (CD56 and CD36), and CD4^+^ T cells (CD4 and FOXP3) across CP5 and CP6 in CRC and GC samples. Scale bars of each group, 100 μm (left) and 50 μm (right). (D) Fluorescence quantification for selected markers was performed across multiple fields of view per sample. Statistical analysis was performed using unpaired Student’s *t* test; *P < 0.05; ****P < 0.0001.

Last, we analyzed and compared the cell–cell communication patterns across 4 CPs (CP1, CP2, CP5, and CP6) associated with liver metastasis using CellPhoneDB [79]. To provide spatial context for these programs, we integrated spatial transcriptomic analyses [80–82] by assessing the spatial enrichment of multigene program signatures derived from scRNA-seq data using UCell-based enrichment scoring (Fig. S6A). Depending on data availability across tumor types, spatial validation was further complemented by single-cell-based spatial deconvolution and multiplex immunofluorescence staining. The overall interaction patterns within each program revealed distinct communication dynamics, shaped by specific immune and stromal cell populations (Fig. 7A).

Specifically, in CP5, robust cellular communication signals were observed between immune cells and endothelial cells, including specialized liver sinusoidal endothelial cells (LSECs) (Fig. 7A). Spatial support was further provided by multiplex immunofluorescence staining in gastric cancer (GC) and colorectal cancer (CRC) cohorts, which revealed enhanced accumulation of NK cells in close proximity to endothelial compartments in early-stage PT samples compared with metastatic tumors (Fig. 7C and D and Fig. S7). While this observation reflects spatial colocalization rather than direct functional ligand–receptor (LR) engagement, it supports the physical plausibility of the predicted NK–endothelial communication inferred from CellPhoneDB analysis. Key interaction pathways activated in this program involved immune cell recruitment via the intercellular adhesion molecule 3–C-type lectin domain family 4 member M (CLEC4M) LR interaction, leukocyte adhesion to the vascular endothelium through the vascular cell adhesion molecule 1–integrin α_4_β_1_ complex, podocalyxin like (PODXL)–selectin L (SELL), and CD34–SELL, as well as apoptosis via TNF superfamily member 10 (TNFSF10)–TNF receptor superfamily member 10b (TNFRSF10B) (Fig. S5C). In addition, the interaction of CD160–TNF receptor superfamily member 14 (TNFRSF14) facilitated the promotion of interferon-γ production. With NK cells (antitumor immune cells) enriched in CP5, they may recruit conventional type 1 dendritic cells (cDC1s) via X-C motif chemokine ligand 2 (XCL2)–X-C motif chemokine receptor 1 (XCR1) for antigen presentation (Fig. 7C and D and Fig. S5D). Consistent with prior studies, the recruitment of cDC1s into tumors by NK-cell-derived chemokines such as XCL1–XCL2 is a recognized mechanism for promoting antitumor immunity [83]. Furthermore, cross-talk between cDC1 and NK cells can reciprocally enhance NK cell function, contributing to antitumor immune surveillance [84]. These findings suggest that CP5 represents a CP within the TME where immune cells begin to actively monitor cancer-cell-derived vascular structures, contributing to immune surveillance.

In CP1, macrophages were central players in cell–cell communication networks within the TME, strongly interacting with epithelial, stromal, and other immune cells (Fig. 7A). CP1 was predominantly enriched in primary liver tumors and pancreatic cancer liver metastases (Fig. 2B). Spatial transcriptomic deconvolution and UCell scoring revealed prominent spatial colocalization of macrophages and epithelial cells in CP1-dominated regions, particularly in PTs (Fig. 7B). This spatial organization was further supported by multiplex immunofluorescence validation in GC and CRC cohorts, which confirmed enhanced macrophage infiltration surrounding epithelial compartments in PTs compared with liver metastases (Fig. S7). CP1 was marked by the activation of macrophage-mediated LR pairs, such as CCL3–CCR1 and retinoic acid receptor responder 2 (RARRES2)–chemerin chemokine-like receptor 1 (CMKLR1) (Fig. S5C), which are associated with immune recruitment and inflammation [85,86]. Notably, in preclinical models of breast cancer pulmonary metastasis, CCL3–CCR1 signaling has been shown to be essential for the retention of metastasis-associated macrophages at the metastatic site, thereby promoting inflammatory remodeling and metastatic niche formation [87]. This established biological function strongly supports the relevance of the CCL3–CCR1 interaction predicted in our CP1 context. Macrophage subtypes such as Mac_STMN1, Mac_SPP1, and Mac_AIF1 promoted angiogenesis and tumor invasion via vascular endothelial growth factor B (VEGFB)–neuropilin 1 (NRP1), fibronectin 1 (FN1)–integrin α_V_β_5_ complex, and fibrillin 1 (FBN1)–integrin α_5_β_1_ complex (Fig. S5D). Macrophages also recruited Tregs through CCL20–CCR6 and influenced endothelial and myofibroblastic cancer-associated fibroblast (myCAF) cells via insulin like growth factor binding protein 3 (IGFBP3)–transmembrane protein 219 (TMEM219), potentially inducing antiproliferative effects. Epithelial cells evaded complement-mediated lysis via the interaction of clusterin (CLU)–triggering receptor expressed on myeloid cells 2 (TREM2) receptor, while NK_GNLY cells were suppressed by immune cells via major histocompatibility complex, class I, C (HLA-C)–killer cell immunoglobulin like receptor, two Ig domains and long cytoplasmic tail 3 (KIR2DL3) interactions. Overall, CP1 was characterized as a macrophage-dominated metastatic TME that promoted tumor angiogenesis, immune suppression, and invasion.

In CP6, strong intercellular communication signals were observed among T cell subtypes, particularly with Tregs exhibiting significant interactions with other immune cells (Fig. 7A). Additional validation with immunofluorescence staining confirmed that forkhead box P3-positive (FOXP3^+^) Tregs exhibited a remarkably enhanced level of interaction with tumor cells in metastatic tumors compared with PTs (Fig. 7C and D and Fig. S7). In CP6, Treg_TNFRSF4 and Treg_BATF may mediate immune suppression within the TME through LR interactions with lymphotoxin alpha (LTA)–TNFRSF1B and LTA–TNFRSF14, regulating immune cell interactions. Meanwhile, exhausted CD8 T (CD8Tex) cells may further enhance Treg activation via TNFSF9–TNFRSF9 and TNFSF4–TNFRSF4 interactions. Th1_GADD45B and Mono_FCN1 may contribute to inflammation-driven carcinogenesis through TNFSF14-mediated cellular interactions (Fig. S5C and D). Overall, CP6 represents a Treg-dominant “immunosuppressive” CP within the TME, where immune regulation is skewed toward promoting immune evasion and fostering an environment conducive to tumor survival and metastasis.

In CP2, there were no notably strong cellular interactions between immune and nonimmune cells, except for the persistent communication between macrophage–monocyte subtypes and epithelial, endothelial, and pericyte cells (Fig. 7A). Consistently, spatial transcriptomic deconvolution and UCell-based scoring revealed the spatial enrichment of CP2-associated macrophage programs and epithelial signatures with limited coordination with other cellular compartments in paired hepatocellular carcinoma (HCC) and pancreatic ductal adenocarcinoma (PDAC) samples, supporting a structurally compartmentalized microenvironment (Fig. 7B). For instance, anti-inflammatory IL-10 signaling pathways were strongly activated in cell subtypes such as Mac_CCL3, Mac_GPR183, Mac_MT1G, and Mono_IL1B, while angiogenesis and tumor growth were promoted in endothelial cells (Fig. 7B and Figs. S5D and S7), suggesting an immunosuppression and immune-desert microenvironment within this program. We confirmed IL-10 expression in endothelial cells (CD31^+^), macrophages (CD68^+^), and tumor cells in both primary and metastatic tumors. Notably, the coexistence of IL-10^+^ cells was significantly increased in metastatic lesions compared to PTs (Fig. S7). Given the presence of enriched epithelial cells in the later stages of the differentiation trajectory (Fig. 3F), CP2 may represent an immunodeficient CP within the TME, where immune surveillance is compromised, facilitating tumor progression and adaptation. This immunosuppressed state in CP2 highlights a potential “immune-excluded” or “immune-desert” TME, characteristic of tumors that are less responsive to immune-based therapies.

## Discussion

**Fig. 8.**
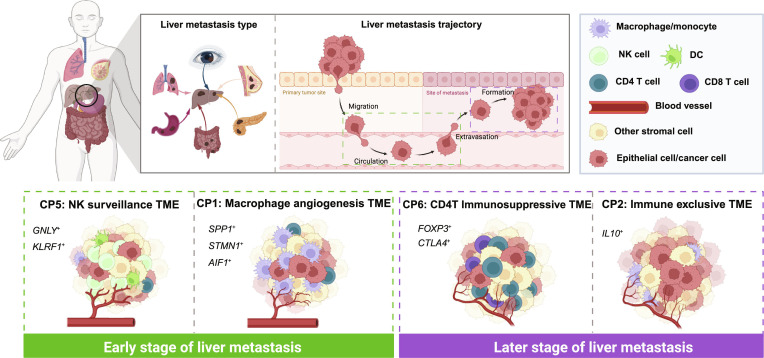
A staged cellular program (CP) model of liver metastatic progression. Schematic illustration of a staged model describing liver metastasis and the evolution of tumor microenvironmental CPs identified in this study. The model is based on integrated single-cell and spatial analyses. Different colors denote major cell types, including epithelial/cancer cells, macrophages/monocytes, natural killer (NK) cells, CD4^+^ T cells, CD8^+^ T cells, dendritic cells (DCs), and other stromal cells, as indicated in the legend. Blood vessels are shown in red. Dashed boxes highlight distinct metastatic stages and their corresponding CPs. CP5 and CP1 represent early-stage, immunoactive, and macrophage-driven angiogenic microenvironments, whereas CP6 and CP2 represent later-stage, immunosuppressive, and immune-excluded states.

In this study, we analyzed 100 scRNA-seq samples comprising over 460,000 cells to generate a comprehensive single-cell transcriptomic landscape of liver metastases across multiple cancer types. By identifying distinct CPs associated with liver metastasis, we revealed how the cellular composition and intercellular interactions within the TME shape metastatic progression. Our findings uncover a complex evolutionary relationship between the CPs, reflecting a dynamic metastatic process in the liver [88], characterized by a transition from immunoactive and inflammatory microenvironments to immunosuppressive and immune-desert states (Fig. 8). This progression highlights how distinct CPs arise in response to evolving immune pressures and stromal cues within the TME at different stages of tumor development. CP5 and CP1 represent early stages of metastasis, where immune cells actively engage with tumor cells and vasculature. In contrast, CP6 and CP2 reflect later stages where immune evasion and suppression dominate. Notably, each CP includes both primary and metastatic samples, supporting a parallel model of tumor evolution [1], at least for certain tumor types, in which cancer cells disseminate early and evolve independently.

CP5, enriched with NK and CD8^+^ T cells, exhibited strong immune–endothelial interactions, indicative of an environment where immune surveillance is still active. The presence of cDC1–NK cell cross-talk via XCL2–XCR1 further suggests that CP5 may represent an intermediate or premetastatic state, where antitumor immunity is engaged but may ultimately be suppressed or evaded as metastases progress. This observation aligns with a notion that functional NK cell activity may be critical for restraining early metastatic outgrowth [89]. However, tumors frequently develop mechanisms to neutralize this immune pressure.

In contrast, CP1 is defined by a macrophage-centric communication network that drives liver metastasis through the activation of specific LR pairs, such as CCL3–CCR1 and RARRES2–CMKLR1. These interactions promote tumor angiogenesis, invasion, stromal remodeling, and the establishment of an immunosuppressive inflammatory microenvironment. Moreover, the enrichment of Treg-recruiting signals such as CCL20–CCR6 further suggests that CP1 fosters an immune-evasive niche, consistent with previous reports linking macrophage–Treg cross-talk to enhanced metastasis [90]. The macrophage-dominated nature of CP1 underscores its pivotal role in promoting metastasis, indicating that targeting macrophages may offer a potential strategy to disrupt tumor growth and metastasis.

In CP6, a Treg-dominated immunosuppressive landscape emerged, where high Treg infiltration and extensive immunosuppressive interactions (e.g., LTA–TNFRSF1B and LTA–TNFRSF14) likely contributed to tumor persistence [91,92]. The presence of CD8Tex cells further suggests that chronic antigen exposure and an immunosuppressive TME drive T cell exhaustion, a hallmark of metastatic adaptation [92–95]. Importantly, our results indicate that Th1 and Th17 cells may actively contribute to this suppressive environment, revising the conventional understanding that these subsets primarily function as antitumor effectors [96,97]. Instead, their interactions with Tregs and monocytes in CP6 suggest a role in reinforcing immune tolerance. This insight has important implications for immunotherapy strategies, highlighting the need to address Treg-driven immune suppression and T cell exhaustion in advanced metastatic cancers to overcome immune resistance. Although Th1 and Th17 cells are classically associated with proinflammatory and antitumor responses, emerging evidence suggests that their functions can shift under chronic inflammatory or tumor-associated conditions. In our study, the Th1_GADD45B and Th17_AREG subtypes showed selective enrichment and preferential interaction with Treg populations within CP6, suggesting a phenotypic and functional transition toward immunoregulatory roles. This observation highlights the plasticity of T helper cell states within the metastatic TME and underscores the complexity of immune modulation during liver metastasis. Future studies will be needed to validate these roles in vivo and to determine the molecular mechanisms driving this potential functional reprogramming.

CP2 is characterized by weak immune interactions, with macrophage and monocyte subtypes interacting with epithelial, endothelial, and pericyte cells. This immune-excluded state reflects impaired immune infiltration and responsiveness to tumor cells. The activation of immunosuppressive pathways points to a lack of immune surveillance, facilitating tumor progression in an “immune-desert” TME. Therefore, CP2 is crucial for understanding tumors that are resistant to immune therapies, emphasizing the need for alternative approaches to overcoming this immunosuppressive environment.

Our findings also refine and extend current models of metastatic progression by demonstrating that distinct CPs reflect different stages of immune engagement and tumor adaptation. While prior studies have largely focused on the intrinsic properties of disseminated tumor cells, our results emphasize that the metastatic outcome is equally dependent on the evolving cross-talk between tumor, immune, and stromal populations. Moreover, the identification of specific LR interactions unique to each CP provides a framework for context-specific targeting of metastasis-promoting mechanisms.

A limitation of this study is that the atlas was constructed entirely from publicly available scRNA-seq datasets. Although this approach enabled integration across diverse tumor types and effective use of existing resources, it cannot fully substitute for newly generated datasets. The number of liver metastasis samples included (*n* = 25) is relatively modest, with uneven distribution across cancer types, which may limit statistical power for detecting rare but potentially critical cellular subsets and constrain the generalizability of cancer-type-specific patterns. In addition, metastases from certain less common cancers (e.g., cholangiocarcinoma and gallbladder cancer) are not represented. Notably, the CPs identified here were derived from integrative analyses across multiple datasets and tumor origins, capturing recurrent microenvironmental states that are shared across liver metastases rather than being driven by individual cancer types. Nevertheless, this study provides the first unified single-cell reference of liver metastases across multiple cancer types, creating a resource that enables systematic cross-cancer comparisons of the metastatic niche and establishes a foundation for future mechanistic and translational investigations.

Importantly, the CP framework established here provides a basis for stratifying patients with liver metastases for immunotherapy. Each CP reflects a distinct immune contexture that may predict therapeutic responsiveness. CP5, enriched for NK cells and CD8Tem cells, represents an immunoactive microenvironment and may identify patients likely to benefit from immune checkpoint blockade or NK-cell-based therapies, particularly at early metastatic stages. CP6 exhibits a Treg-dominated, immunosuppressive landscape with CD8^+^ T cell exhaustion. Checkpoint inhibitors alone may therefore be insufficient, and combination strategies targeting Tregs, reversing T cell exhaustion, or disrupting immunosuppressive cytokines may be required. CP1, defined by macrophage-centric communication and protumor LR interactions, highlights a myeloid-driven metastatic niche and indicates potential benefit from therapies targeting macrophage recruitment, polarization, or macrophage–Treg cross-talk. CP2 represents an immune-cold, immune-excluded state with limited immune infiltration, commonly associated with checkpoint blockade resistance; patients in this program may require immune-priming strategies such as stromal modulation or vascular normalization. Collectively, these observations demonstrate that CP-based classification captures clinically relevant immune states and provides a concrete rationale for patient stratification and tailored immunotherapy approaches in liver metastases.

In summary, this study presents a refined view of liver metastasis as a dynamic process shaped by evolving intercellular interactions. By defining recurrent CPs across liver metastasis samples, we demonstrate that metastatic progression is accompanied by systematic shifts in immune engagement, stromal remodeling, and tumor-immune cross-talk. Importantly, the CP framework provides an integrative and clinically relevant perspective that links tumor microenvironmental states with therapeutic vulnerabilities. This conceptual framework may facilitate patient stratification, guide rational immunotherapy design, and support the identification of context-dependent therapeutic strategies for metastatic cancer.

## Materials and Methods

### scRNA-seq data collection, processing, and integration

scRNA-seq data were obtained from publicly available datasets, with all samples generated using the 10x Genomics platform. Raw gene count matrices were aligned to the GRCh38 reference genome. Filtered count matrices were converted to sparse matrices using the Seurat package (version 4.2.0) [98], and cells expressing fewer than 200 genes or containing >20% mitochondrial reads were excluded from downstream analyses. The DoubletFinder package (version 2.0.3) [99] was used using the “doubletFinder_v3” function to identify and remove potential doublets.

Following quality control, data were normalized using SCTransform, regressing out unique molecular identifier (UMI) counts and mitochondrial transcript percentages. To mitigate batch effects across samples and experiments, we applied Seurat’s SCTransform-based integration workflow with reciprocal principal components analysis. A total of 2,000 integration features were selected using the “SelectIntegrationFeatures” function. Integration anchors were identified using the “FindIntegrationAnchors” function with reciprocal principal components analysis reduction, based on 30 principal components (k.anchor = 10). Default anchor filtering parameters were used. The integrated expression matrix was generated using IntegrateData with 30 principal components.

### Cell clustering and identification

Cell clustering was conducted using the “FindClusters” function, with a resolution of 0.8 applied during the first integration and 2.0 during the second integration. The top 20 differentially expressed genes were used to define cell identities. Dimensionality reduction was performed using the “RunUMAP” function, and results were visualized using UMAP.

In our study, 3 hierarchical levels of cell classification were identified, encompassing 19 major cell types, 34 subset cell types, and 121 subgroups. Major and subset cell types were delineated on the basis of established marker genes (Fig. S1B and C). For subgroup clustering, cells of different types were individually extracted and clustered on the basis of their respective first 30 principal components, with clustering resolutions adjusted according to visual inspection.

To identify marker genes characteristic of each cell subset and subgroup, the log_2_ fold change between each cluster (subset or subgroup) and all other cells was computed using the “FindMarkers” function with the Wilcoxon rank-sum test and default parameters. Expression heatmaps of identified marker genes were generated using the ComplexHeatmap R package (version 2.6.2) [100] after normalization (Fig. 1E).

To enhance the biological credibility of our annotations, we systematically cross-referenced the identified subsets with multiple high-quality reference atlases, including the Human Cell Atlas, Tumor Immune Cell Atlas, and TISCH, as well as several large-scale pan-cancer single-cell studies. These references provided robust benchmarks for annotating both canonical and context-specific immune, stromal, and epithelial populations across cancer types.

### Spatial transcriptomic data processing and annotation

Spatial transcriptomic data were obtained from public datasets across multiple cancer types, including CRC, PDAC, and breast cancer. Raw spatial transcriptomic data in the 10x Genomics format were processed using the Seurat package (version 4.2.0). For each sample, expression matrices and spatial image files were loaded and preprocessed. Spots were filtered and normalized using the SCTransform method.

To annotate spatial regions with putative cell types, we utilized a curated list of top 20 marker genes per cell subtype identified from scRNA-seq data. AUCell, a gene set enrichment scoring method designed for single-cell applications, was applied to compute enrichment scores for each marker gene set across spatial transcriptomic spots. This approach enabled probabilistic assignment of cell type identities based on transcriptional similarity to scRNA-seq-defined cell states.

### Clustering based on pseudo-bulk expression

Pseudo-bulk gene expression profiles were generated using the “AverageExpression” function. Transcriptional relationships among the 121 subgroups were assessed by hierarchical clustering based on Spearman correlation, computed using the “cor” function. The resulting dendrogram was extracted using the dendextend package (version 1.15.2) [101] and visualized with the pheatmap package (version 1.0.12) [102].

### Clustering and definition of CPs

Cellular infiltration rates were quantified for each liver-related sample, defined as the proportion of cells belonging to each of 34 major cell types among all cells in a given sample. This resulted in a sample-by-cell-type matrix representing cellular composition profiles.

Pairwise similarities between samples were computed using Spearman correlation based on their cellular infiltration profiles. The resulting sample–sample correlation matrix was subjected to unsupervised hierarchical clustering with complete linkage.

CPs were defined by partitioning the hierarchical clustering dendrogram into 6 major clusters based on the separation and stability of dominant branches. Each CP represents a recurrent cellular composition pattern shared across samples. Importantly, CPs were identified in a fully data-driven manner and were independent of cancer type, tissue origin, or clinical annotations.

Subsequent analyses were performed to characterize the cellular composition, functional features, and clinical relevance of each CP.

### CNV estimation in epithelial cells

CNVs in epithelial cells were inferred using the InferCNV package (version 1.6.0) [63] with default parameters to identify potential cancer cells. CNV signals for individual cells were estimated using a sliding window of 100 genes. Prior to analysis, genes with a mean expression count below 0.1 across all cells were excluded. The CNV signal was subsequently denoised by applying a dynamic threshold of 1.3 standard deviations from the mean. Epithelial cells were clustered into 66 groups based on CNV accumulation scores using the “FindClusters” function.

### Enrichment analysis

Differentially expressed genes were detected by the “FindAllMarkers” function in Seurat, with a threshold of |log fold change| > 0.25 and an adjusted *P* < 0.05. Gene set enrichment analysis of differentially expressed genes was conducted using the clusterProfiler package (version 4.3.2.991) [103], referencing the hallmark gene sets and C5 (ontology gene sets) collections in the MSigDB database. Differences between cell groups were assessed using the “FindMarkers” function in the Seurat package. Functional scores of macrophages, monocytes, NK cells and CD4^+^ T cells were computed using the UCell package (version 1.99.7) [73] and using known cell type functional markers [45,104,105].

### CP distribution of cell clusters

To characterize the distribution preference of individual cell clusters across CPs, the ratio of observed to expected cell numbers (*R*_o/e_) [71,72] was calculated for each cell cluster in different CPs to quantify the CP preference of each cell cluster. The expected cell numbers for each combination of cell clusters and CPs were obtained from the chi-square test. One cluster was identified as being enriched in a specific CP if *R*_o/e_ > 1.

### Pseudotime analysis

The developmental trajectories of epithelial cells, macrophages, monocytes, and CD4^+^ T cells were inferred using Monocle2 (version 2.18.0) [67] with default parameters as recommended by the developers. First, integrated gene expression matrices from specific cell types were exported from Seurat into Monocle to construct a CellDataSet. Next, core dataset information was computed using the “estimateSizeFactors”, “estimateDispersions”, and “detectGenes” functions. Variable genes were identified using the “differentialGeneTest” function and subsequently used to order cells via the “setOrderingFilter” function. Finally, dimensionality reduction was performed using the “reduceDimension” function with the “DDRTree” reduction method, and key regulatory genes involved in differentiation were visualized along the trajectory using the “plot_pseudotime_heatmap” function.

To determine the root of differentiation within epithelial cell clusters, CytoTRACE (version 0.3.3) [66] was applied to predict differentiation potential. The root of the developmental trajectory was manually assigned on the basis of prior biological knowledge and CytoTRACE predictions. Following root cell determination, cells were reordered along the trajectory using the “order_cells” function of Monocle2.

### Survival analysis

RNA-seq expression profiles and corresponding clinical information of patients with LIHC were obtained from TCGA to evaluate the prognostic relevance of macrophage-associated gene signatures. Only PT samples were included in the analysis. Overall, survival time was defined as the interval from initial diagnosis to death or last follow-up, and survival status was coded as event (death) or censoring (alive).

To quantify the activity of macrophage-subtype-specific gene programs in bulk tumors, gene set variation analysis (GSVA) was performed using the GSVA R package (version 1.38.2) [106]. For each macrophage/monocyte subtype, gene sets were constructed by selecting the top differentially expressed marker genes (typically the top 20 genes ranked by average log_2_ fold change; 30 genes were used for Mac_CCL18 due to its broader marker coverage). GSVA enrichment scores were calculated for each tumor sample based on normalized RNA-seq expression data.

Tumor samples were stratified into high and low groups according to the mean GSVA score of each gene set. For macrophage subtypes showing sufficient score variability, an optimal cutoff was additionally determined using maximally selected rank statistics implemented in the survminer package; otherwise, mean-score-based dichotomization was applied.

Kaplan–Meier survival curves were generated using the survival R package (version 3.2.13) [107] and visualized with the “ggsurvplot” function from the survminer package (version 0.4.9). [108]. Numbers of patients at risk were displayed below each survival curve to indicate the temporal distribution of evaluable samples. Statistical differences between survival curves were assessed using a 2-sided log-rank test. A *P* < 0.05 was considered statistically significant.

### Cell–cell interaction analysis

Cell–cell interactions among different cell types were inferred using CellPhoneDB (version 5.0.0) [79] with default parameters (requiring at least 20% of cells to express either the ligand or receptor). CellPhoneDB predicts potential interaction strength between 2 cell subsets based on the gene expression level of an LR pair. Statistical significance was evaluated using a permutation test (1,000 to 5,000 iterations), with normalized gene expression data as input. Interactions with *P* < 0.05 were considered significant.

### Spatial transcriptomic deconvolution

To validate the cellular composition of CP1/CP2-dominated tumor ecosystems in liver and pancreatic cancers, we performed spatial transcriptomic deconvolution on paired HCC and PDAC samples. The datasets included matched normal and tumor regions from HCC, as well as paired PTs and liver metastases from PDAC.

Cell type deconvolution was carried out using robust cell type decomposition, with an integrated scRNA-seq dataset serving as the reference. Raw UMI count matrices were extracted from the RNA assay, gene identifiers were harmonized to gene symbols, and duplicated genes were aggregated. Cell type annotations and per-cell UMI counts were used to construct the robust cell type decomposition reference object.

For each spatial transcriptomic sample, raw spatial counts and tissue coordinates were extracted from the spatial assay to generate query objects. Deconvolution was performed using the doublet mode to account for mixed-cell spots. Cell type proportion weights were normalized and used for downstream spatial visualization and comparative analyses between normal and tumor regions in HCC and between primary and metastatic lesions in PDAC.

### Spatial-transcriptomics-based validation of CP-associated CPs

Cell-type-specific gene signatures were derived from scRNA-seq differential expression analyses. For each annotated cell subtype, genes with an average log_2_ fold change > 0.5 and an adjusted *P* < 1 × 10^−4^ were ranked by effect size, and the top 500 genes were selected to define subtype-specific gene sets. This gene set size was chosen to balance robustness and specificity, particularly in the context of low-resolution spatial transcriptomics data. For epithelial cells, endothelial cells, pericytes, and myCAFs, canonical marker genes were additionally included to enhance biological interpretability such as epithelial (e.g., *EPCAM*, *KRT18*, *CD24*, *KRT19*, *PAX8*, *SCGB2A2*, *KRT5*, and *KRT15*), endothelial (*CLDN5*, *PECAM1*, *VWF*, *FLT1*, and *RAMP2*), pericyte (*NOTCH3*, *CD146*, and *RGS5*), and myCAF (*RGS5*, *ACTA2*, and *TAGLN*).

Cell type enrichment scores in spatial transcriptomics data were calculated using the UCell algorithm, a rank-based gene set scoring method that is robust to technical variability and sequencing depth differences. UCell scores were computed at the spot level using the “AddModuleScore_UCell” function applied to the SCTransform-normalized assay. These enrichment scores were used for downstream spatial visualization and comparative analyses across samples.

### Multiplexed immunofluorescence staining

Formalin-fixed, paraffin-embedded tissue specimens were obtained from the Department of Pathology, Nanjing Drum Tower Hospital, the Affiliated Hospital of Nanjing University Medical School. The study cohort included the following:

• Early-stage primary GC: 4 cases
• Early-stage primary CRC: 4 cases
• Colorectal adenocarcinoma with synchronous HMs (CC-HM): 8 cases (matched primary/metastatic lesions)
• Gastric adenocarcinoma with synchronous HMs (GC-HM): 6 cases (matched primary/metastatic lesions)

Multiplex immunofluorescence was performed on PT and HM specimens using the PANO 5-plex IHC kit (catalog no. 10002100100, Panovue, Beijing, China) according to the manufacturer’s instructions. Multiple primary antibodies, including CD68, secreted phosphoprotein 1 (SPP1), stathmin 1 (STMN1), pan-cytokeratin, CD56, CD31, CD36, CD4, FOXP3, and IL-10, were used in this study (Table S2), followed by incubation with horseradish-peroxidase-conjugated secondary antibodies and tyramide signal amplification. The slides were microwave-heat-treated after each tyramide signal amplification operation. Nuclei were stained with 4′,6-diamidino-2-phenylindole (DAPI) after all the antigens above had been labeled. The stained slides were imaged at ×10 magnification using a Vectra 3.0 Automated Quantitative Imaging System (PerkinElmer), and regions of interest were selected for multispectral image acquisition at ×20. Five high-power fields were taken per patient sample to quantitate the average number of certain cell populations.

### Multiplex quantification

Relative fluorescence intensity for each marker was quantified using ImageJ by measuring the mean intensity in defined regions of interest per field of view. Three independent fields per sample were analyzed. Graphs were plotted in GraphPad Prism 8.0.2, and statistical significance was determined using unpaired one-tailed Student’s *t* test. Specific *P* values were labeled in the plots or figure legends, where significant values were *P* < 0.05. All data in graphs were presented as means ± SD. No data were excluded from the analyses presented in this study.

### Statistical analysis

Statistical analyses and data visualization were conducted in R (version 4.0.0) [109], and various R packages were used for specific tasks. Graphics were created using packages such as ggplot2 (version 3.4.4) [110] and ggsankey (version 0.0.9) [111]. If not otherwise stated, all statistical procedures were carried out within the R environment.

## Data Availability

The raw scRNA-seq and spatial transcriptomic data used in this study were obtained from publicly available databases. Specifically, data were retrieved from the Gene Expression Omnibus under accession number GSE139829, GSE158803, GSE178318, GSE140312, GSE149614, GSE138709, GSE179994, GSE123902, GSE169246, GSE176078, GSE197177, GSE154778, GSE162708, GSE163558, GSE225857, GSE272362, GSE274557, and HRA000437; from the National Genomics Data Center under accession number OMIX001073 (https://ngdc.cncb.ac.cn/); and from the Tabula Sapiens dataset (https://tabula-sapiens.sf.czbiohub.org/), which is referred to as the Science_normal project in Table S1. The processed scRNA-seq data generated in this study are available at https://biobigdata.nju.edu.cn/scPLM/. The TCGA dataset was obtained from the GDC data portal (https://portal.gdc.cancer.gov/). For datasets obtained from the Human Tumor Atlas Network (HTAN; https://humantumoratlas.org/), including HTAN_WUSTL_Breast and HTAN_WUSTL_CRC, additional datasets used in this study are available within the article, Supplementary Materials, or source data file. This study analyzed publicly available scRNA-seq datasets. No new human tissue samples were collected or used, and all original data were generated under appropriate ethical approvals as reported in the source publications. All codes used to analyze data and generate figures have been uploaded to the GitHub (https://github.com/compbioNJU/).
