# Supplementary material for: A Single-Cell Atlas of Pan-Cancer Liver Metastasis Reveals Dynamic Cellular Programs Driving Metastatic Progression and Immune Modulation
Source: Research (Wash D C). 2026 Mar 24;9:1208. doi: 10.34133/research.1208 (PMC13010057; doi:10.34133/research.1208)
Supplement: Supplementary 1 — Figs. S1 to S7 Tables S1 and S2 [file research.1208.f1.zip › 2minor_Revised Research_supplementary_materials.docx]

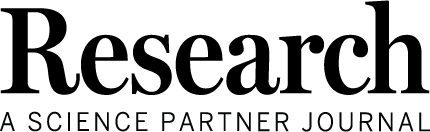


Supplementary Materials for

**A single-cell atlas of pan-cancer liver metastasis reveals dynamic cellular programs driving metastatic progression and immune modulation**

Xinyu Tong *et al.*

*Corresponding author. Email: dijunchen@nju.edu.cn

Supplementary


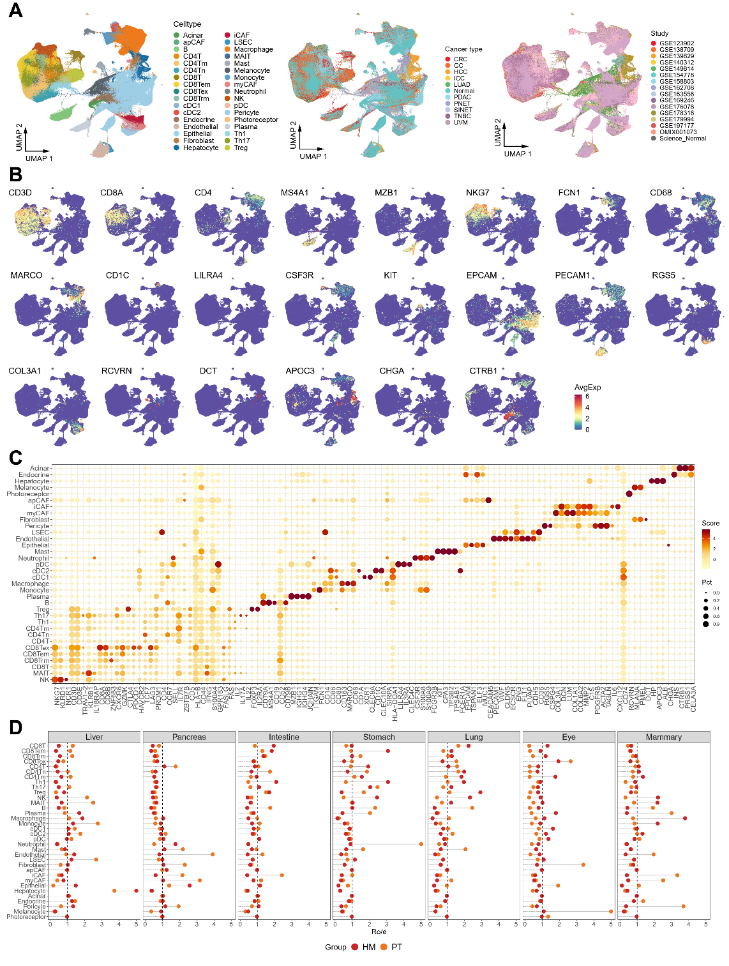


*Fig. S1.*

***A detailed cellular landscape of pan-cancer liver metastasis at the single-cell transcriptomic level. Related to Figure 1.*** ***(A)*** *UMAP plots of all cells colored by major cell types (left), cancer types (middle), and study sources (right), demonstrating effective integration across datasets after batch correction.* ***(B)*** *UMAP plot showing the average expression of key marker genes for major cell types across all cells.* ***(C)*** *Dot plot showing the average expression of key marker genes for major subset cell types. Dot color and size represent average expression level and percentage of cells expressing the marker.* ***(D)*** *Dumbbell plot showing the proportion of each major subset cell type in primary tumors and liver metastases across different organ origins.*


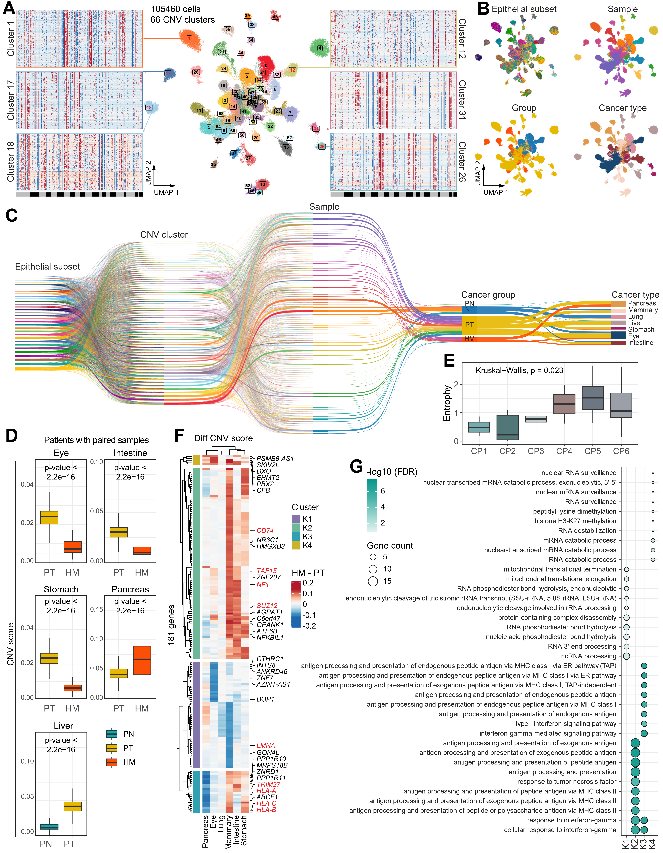


*Fig. S2.*

***CNV heterogeneity of epithelial cells.*** ***(A)*** *UMAP plot displaying all epithelial cells clustered based on CNV score. Heatmap showing CNV inferred by scRNA-seq in CNV cluster 1, 12, 17, 18, 26, 31 cells.* ***(B)*** *UMAP plots showing sample origin, health/cancer status, and cancer type of clustered cells.* ***(C)*** *Sankey diagram illustrating the composition of health/cancer status and cancer types in CNV score-based clusters.* ***(D)*** *Boxplots comparing CNV scores between liver metastases and their matched primary tumors across different organ origins. The boxes indicate the median (horizontal line), interquartile range (box), and Tukey-style whiskers. P values were calculated using one-sided Wilcoxon rank-sum test. *P < 0.05.* ***(E)*** *Boxplot comparing the entropy scores of epithelial cells across different cellular programs. The P value was calculated with Kruskal-Wallis test. *P < 0.05.* ***(F)*** *Heatmap showing hierarchical clustering of gene CNV score differences between liver metastases and primary tumors from different organ origins.* ***(G)*** *Dot plot displaying top 10 Gene ontology analysis performed separately for each gene group (K1–K4) of the clusters identified in F using clusterProfiler. The size and color of the dot represent the gene count enriched and P values from one-sided Fisher’s exact test, adjusted with the Benjamini-Hochberg method. *P < 0.05.*


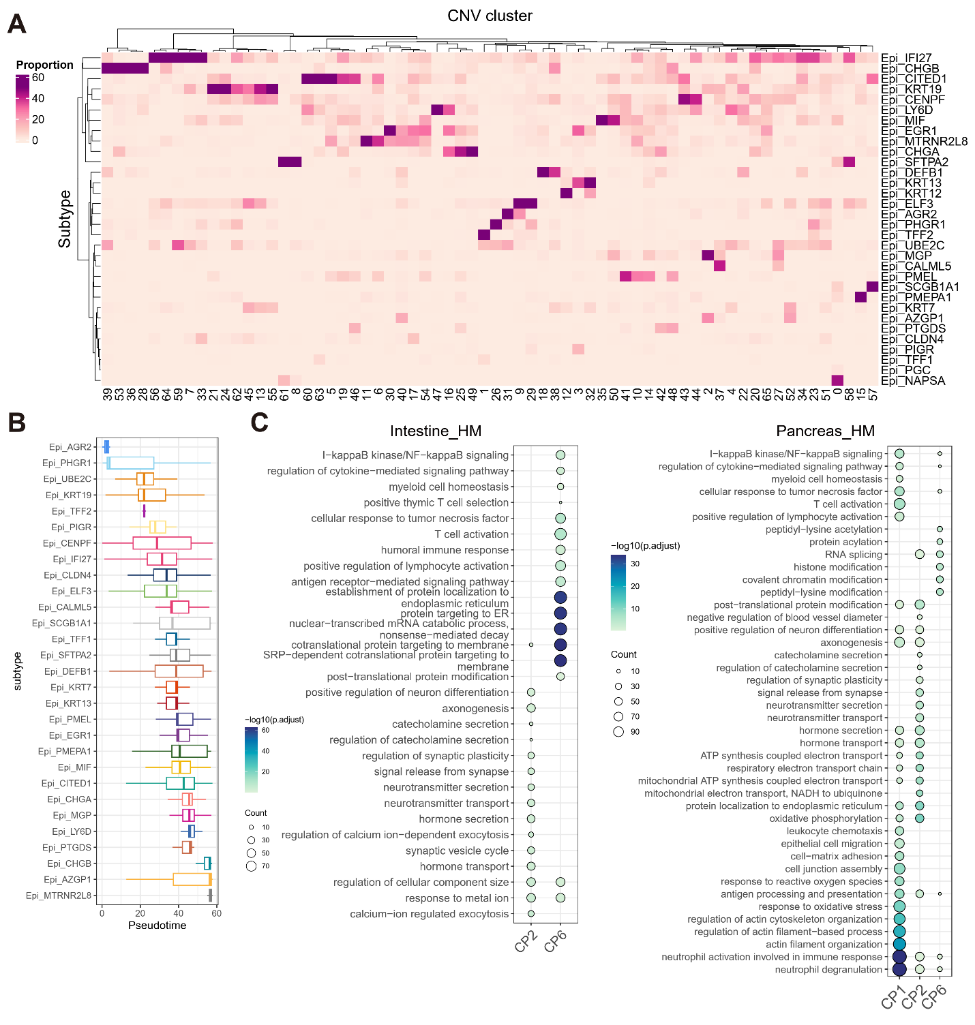


*Fig. S3.*

***Detailed characteristics of epithelial cells. Related to Figure S2 and Figure 4.*** ***(A)*** *Heatmap showing the CNV cluster proportion distribution of each epithelial subtype.* ***(B)*** *Boxplot showing pseudotime scores of epithelial cell subpopulations.* ***(C)*** *Dot plots displaying Gene ontology analysis of the epithelial cells in intestine liver metastases and pancreas liver metastases using clusterProfiler. The size and color of the dot represent the gene count enriched and P values from one-sided Fisher’s exact test, adjusted with the Benjamini-Hochberg method, respectively. *P < 0.05.*


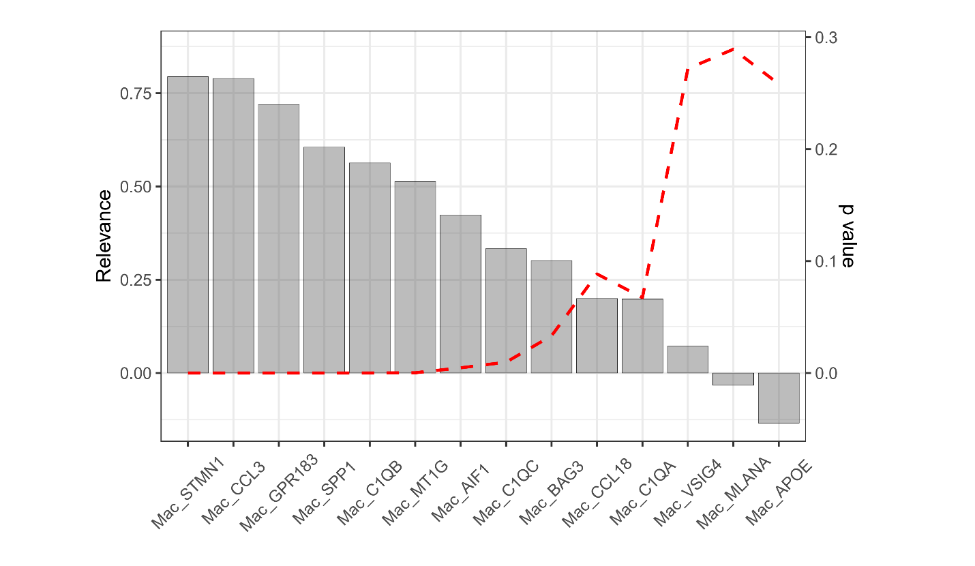


*Fig. S4.*

*Related to Figure 5. Relevance between infiltration rates of macrophage in TME and infiltration rates of each macrophage/monocyte subtype. The bar and red dotted line represent relevance and P value, respectively. *P < 0.05.*


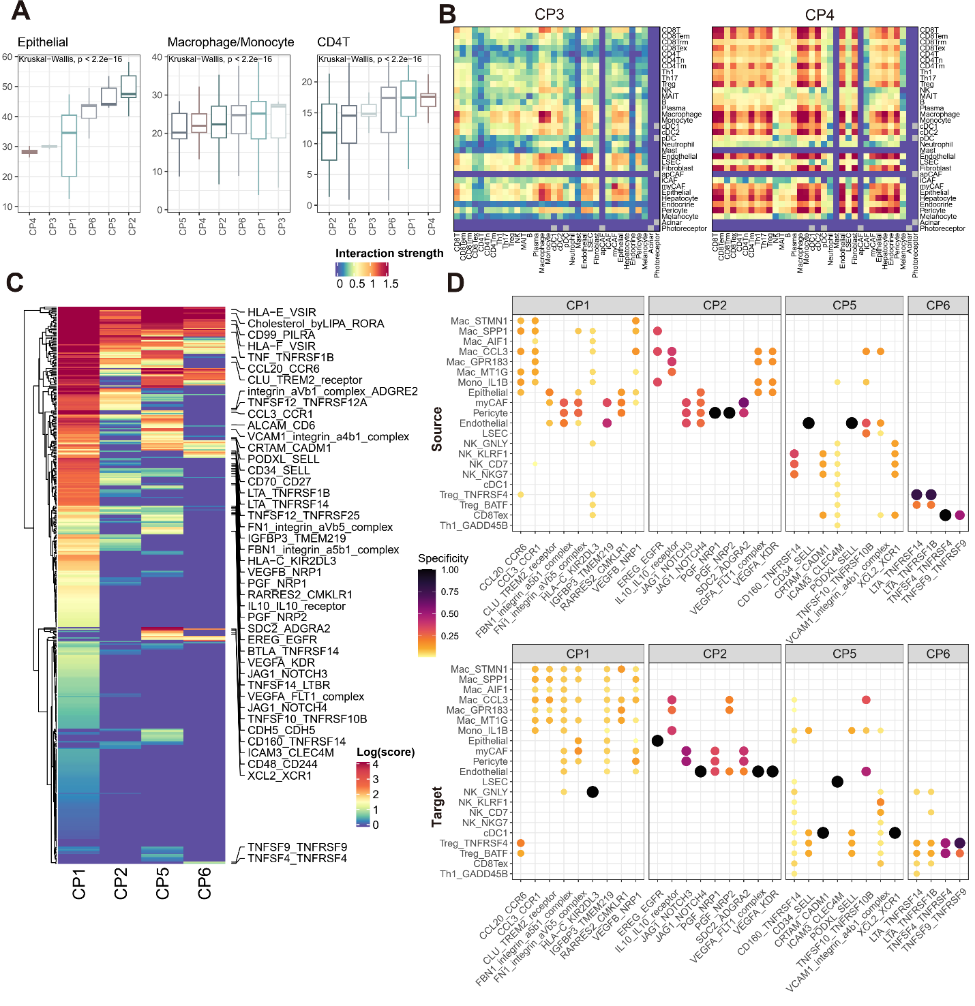


*Fig. S5.*

***Detailed characteristics of Cell–cell interaction networks in the tumor environment within different cellular programs. Related to Figure 7.*** ***(A)*** *Boxplots showing the pseudotime score of epithelial cells, macrophages/monocytes and CD4+T cells in different cellular programs.* ***(B)*** *Heatmaps illustrating the cell–cell interaction patterns in CP3 and CP4.* ***(C)*** *Heatmap showing the interaction scores for significant ligand–receptor pairs in CP1, CP2, CP5 and CP6, respectively.* ***(D)*** *Dot plots showing the enrichment of CP-specific ligand–receptor pairs in key cell subtypes.*


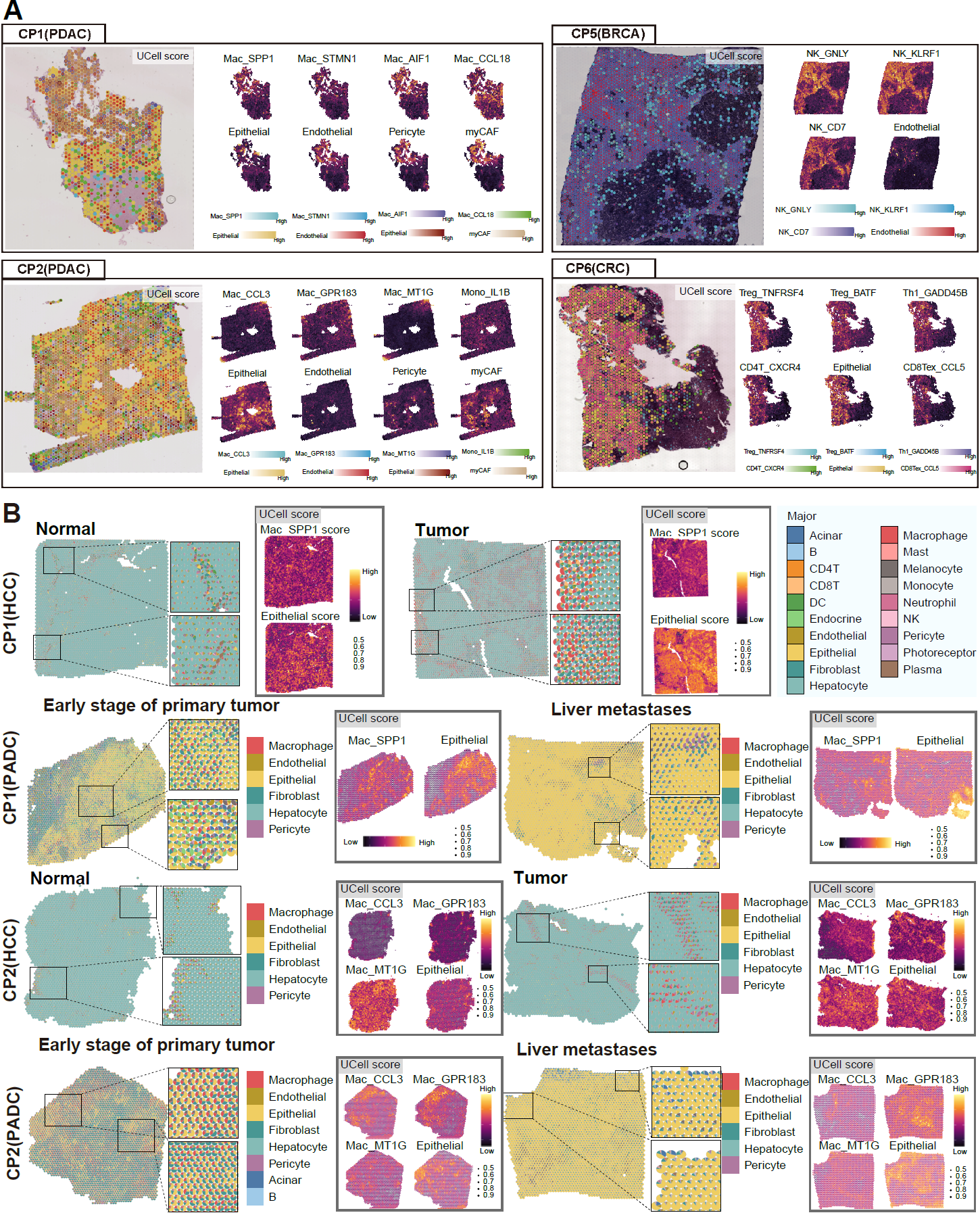


*Fig. S6.*

***Spatial mapping and validation of cell type distribution across primary and metastatic tumor sites. Related to Figure 7.*** ***(A)*** *Spatial mapping of cell-type–specific gene signature enrichment across spatial transcriptomics slides from multiple cancer types corresponding to CP1, CP2, CP5, and CP6. Cell type enrichment was quantified using UCell scores calculated from subtype-specific gene signatures derived from scRNA-seq data. Spatial transcriptomic data were derived from PDAC, BRCA, and CRC samples. The left panel shows the color scale used to represent normalized UCell enrichment scores, while the right panels display the spatial distribution of enrichment scores for each individual cell type.* ***(B)*** *Spatial validation of CP1- and CP2-associated macrophage programs and epithelial signatures in HCC and PDAC samples using single-cell–based spatial deconvolution and UCell scoring. Color intensity and point size represent relative enrichment.*


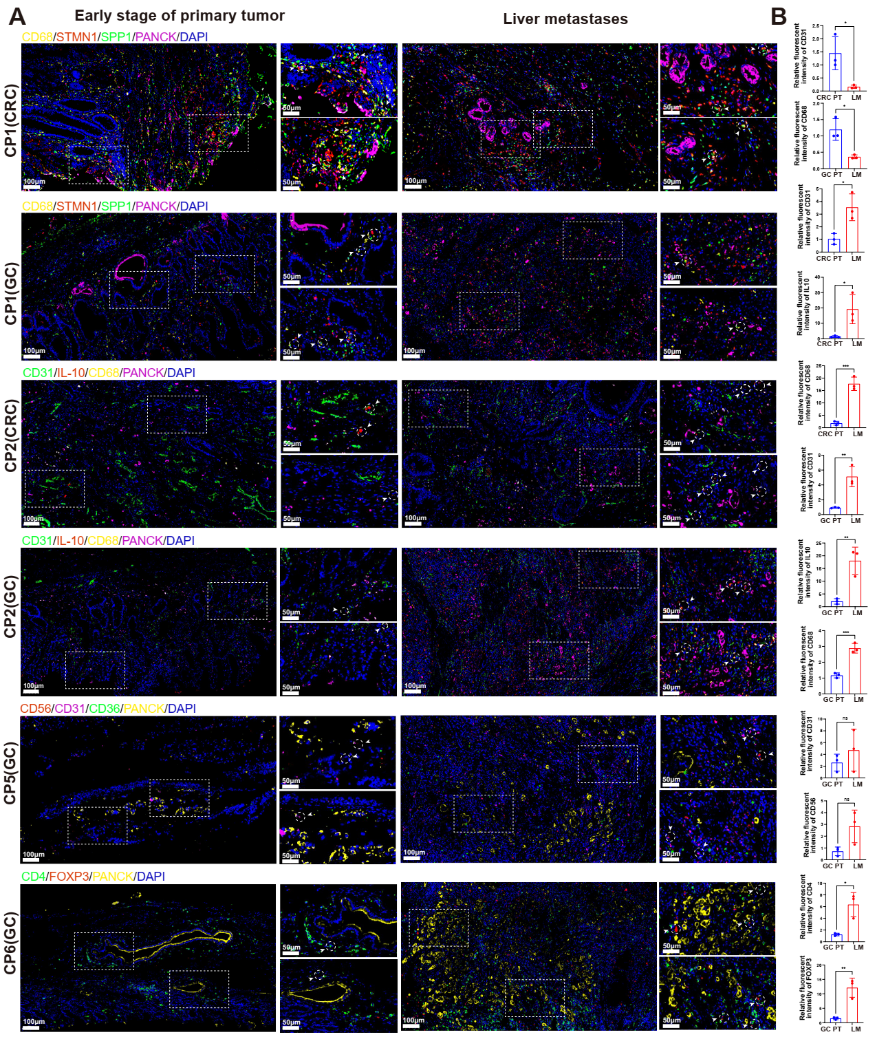


*Fig. S7.*

***Multiplex immunofluorescence analysis of key cellular program–associated markers in primary tumors and liver metastases. Related to Figure 7. (A)*** *Representative immunofluorescence images showing marker expression patterns for tumor epithelial cells (PANCK), macrophages (CD68, STMN1, SPP1), endothelial cells (CD31), immune-suppressive signals (IL-10), NK cells (CD56, CD36), and CD4+ T cells (CD4, FOXP3) across CP1, CP2, CP5, and CP6 in CRC and GC samples. Scale bars: 100 μm (left) and 50 μm (right).* ***(B)*** *Fluorescence quantification for selected markers was performed across multiple fields of view per sample. Statistical analysis was performed using two-tailed Student’s t-test; *P < 0.05.*

*Table S1.*

*Basic characteristics of involved patients.*

| **Project** | **Sample** | **SampleID** | **Cancer Type** | **Stage** | **Tissue** | **Stage** | **Grade** | **TNM** |
| --- | --- | --- | --- | --- | --- | --- | --- | --- |
| Science_Normal | Eye_10X_TSP15 | Eye01-NN | Normal | NA | Eye | NA | NA | NA |
| Science_Normal | Eye_10X_TSP3 | Eye02-NN | Normal | NA | Eye | NA | NA | NA |
| Science_Normal | Eye_10X_TSP5 | Eye03-NN | Normal | NA | Eye | NA | NA | NA |
| GSE139829 | GSM4147091 | UVM01-HM | UVM | NA | Eye | 1B（gep class） | NA | NA |
| GSE139829 | GSM4147092 | UVM02-HM | UVM | NA | Eye | 2（gep class） | NA | NA |
| GSE139829 | GSM4147100 | UVM03-HM | UVM | NA | Eye | 2（gep class） | NA | NA |
| GSE158803 | GSM4810688 | UVM04-HM | UVM | NA | Eye | NA | NA | NA |
| GSE139829 | GSM4147093 | UVM04-PT | UVM | NA | Eye | 2（gep class） | NA | NA |
| GSE158803 | GSM4810690 | UVM05-HM | UVM | NA | Eye | NA | NA | NA |
| GSE139829 | GSM4147094 | UVM05-PT | UVM | NA | Eye | 2（gep class） | NA | NA |
| GSE139829 | GSM4147095 | UVM06-PT | UVM | NA | Eye | 1A（gep class） | NA | NA |
| GSE139829 | GSM4147097 | UVM07-PT | UVM | NA | Eye | 2（gep class） | NA | NA |
| GSE139829 | GSM4147098 | UVM08-PT | UVM | NA | Eye | 1A（gep class） | NA | NA |
| GSE139829 | GSM4147099 | UVM09-PT | UVM | NA | Eye | 2（gep class） | NA | NA |
| GSE178318 | GSM5387661 | CRC01-HM | CRC | IVA | Intestine | IVA | NA | T4aN1cM1a |
| GSE178318 | GSM5387660 | CRC01-PT | CRC | IVA | Intestine | IVA | NA | T4aN1cM1a |
| GSE178318 | GSM5387663 | CRC02-HM | CRC | IVA | Intestine | IVA | NA | T3N0M1a |
| GSE178318 | GSM5387662 | CRC02-PT | CRC | IVA | Intestine | IVA | NA | T3N0M1a |
| GSE178318 | GSM5387668 | CRC03-HM | CRC | IVA | Intestine | IVA | NA | T4bN2bM1a |
| GSE178318 | GSM5387667 | CRC03-PT | CRC | IVA | Intestine | IVA | NA | T4bN2bM1a |
| Science_Normal | Large_Intestine_10X_TSP14 | LI01-NN | Normal | NA | Intestine | NA | NA | NA |
| Science_Normal | Large_Intestine_10X_TSP2 | LI02-NN | Normal | NA | Intestine | NA | NA | NA |
| Science_Normal | Small_Intestine_10X_TSP14 | SI01-NN | Normal | NA | Intestine | NA | NA | NA |
| Science_Normal | Small_Intestine_10X_TSP2 | SI02-NN | Normal | NA | Intestine | NA | NA | NA |
| GSE140312 | GSM4159165 | SINET01-HM | SINET | NA | Intestine | NA | NA | NA |
| GSE140312 | GSM4159164 | SINET01-PT | SINET | NA | Intestine | NA | NA | NA |
| GSE149614 | GSM4505944 | HCC01-PT | HCC | I | Liver | I | NA | T1N0M0 |
| GSE149614 | GSM4505945 | HCC02-PT | HCC | I | Liver | I | NA | T1N0M0 |
| GSE149614 | GSM4505946 | HCC03-PN | HCC | I | Liver | I | NA | T1N0M0 |
| GSE149614 | GSM4505947 | HCC03-PT | HCC | I | Liver | I | NA | T1N0M0 |
| GSE149614 | GSM4505949 | HCC04-PT | HCC | II | Liver | II | NA | T2N0M0 |
| GSE149614 | GSM4505950 | HCC05-PN | HCC | IIIA | Liver | IIIA | NA | T3N0M0 |
| GSE149614 | GSM4505951 | HCC05-PT | HCC | IIIA | Liver | IIIA | NA | T3N0M0 |
| GSE149614 | GSM4505952 | HCC06-PN | HCC | IIIA | Liver | IIIA | NA | T3N0M0 |
| GSE149614 | GSM4505953 | HCC06-PT | HCC | IIIA | Liver | IIIA | NA | T3N0M0 |
| GSE149614 | GSM4505954 | HCC07-PN | HCC | IIIB | Liver | IIIB | NA | T4N0M0 |
| GSE149614 | GSM4505956 | HCC07-PT | HCC | IIIB | Liver | IIIB | NA | T4N0M0 |
| GSE149614 | GSM4505957 | HCC08-PN | HCC | IIIB | Liver | IIIB | NA | T4N0M0 |
| GSE149614 | GSM4505959 | HCC08-PT | HCC | IIIB | Liver | IIIB | NA | T4N0M0 |
| GSE149614 | GSM4505960 | HCC09-PN | HCC | IV | Liver | IV | NA | T4N0M0 |
| GSE149614 | GSM4505961 | HCC09-PT | HCC | IV | Liver | IV | NA | T4N0M0 |
| GSE149614 | GSM4505963 | HCC10-PN | HCC | IV | Liver | IV | NA | T4N0M1 |
| GSE149614 | GSM4505964 | HCC10-PT | HCC | IV | Liver | IV | NA | T4N0M1 |
| GSE138709 | GSM4116579 | ICC01-PN | ICC | III | Liver | NA | III | NA |
| GSE138709 | GSM4116580 | ICC01-PT | ICC | III | Liver | NA | III | T3N1MX |
| GSE138709 | GSM4116582 | ICC02-PN | ICC | III | Liver | NA | III | NA |
| GSE138709 | GSM4116583 | ICC02-PT | ICC | III | Liver | NA | III | T2NXMX |
| GSE138709 | GSM4116584 | ICC03-PT_1 | ICC | III | Liver | NA | II | T2N0M0 |
| GSE138709 | GSM4116585 | ICC03-PT_2 | ICC | III | Liver | NA | II | T2N0M0 |
| Science_Normal | Liver_10X_TSP14 | Liver01-NN | Normal | NA | Liver | NA | NA | NA |
| Science_Normal | Liver_10X_TSP6 | Liver02-NN | Normal | NA | Liver | NA | NA | NA |
| GSE179994 | GSM5444620 | LUAD01-HM | LUAD | NA | Lung | NA | NA | NA |
| GSE179994 | GSM5444631 | LUAD02-HM | LUAD | NA | Lung | NA | NA | NA |
| GSE179994 | GSM5444635 | LUAD03-HM | LUAD | NA | Lung | NA | NA | NA |
| GSE123902 | GSM3516662 | LUAD04-PT | LUAD | IA | Lung | IA | NA | T1bN0M0 |
| GSE123902 | GSM3516663 | LUAD05-PT | LUAD | IA | Lung | IA | NA | T1bN0M0 |
| GSE123902 | GSM3516665 | LUAD06-PT | LUAD | IV | Lung | IV | NA | T2bN1M1 |
| GSE123902 | GSM3516667 | LUAD07-PT | LUAD | IA | Lung | IA | NA | T1bN0M0 |
| GSE123902 | GSM3516672 | LUAD08-PT | LUAD | IB | Lung | IB | NA | T2aN0M0 |
| GSE123902 | GSM3516674 | LUAD09-PT | LUAD | IA | Lung | IA | NA | T1aN0M0 |
| Science_Normal | Lung_10X_TSP1 | Lung01-NN | Normal | NA | Lung | NA | NA | NA |
| Science_Normal | Lung_10X_TSP14 | Lung02-NN | Normal | NA | Lung | NA | NA | NA |
| Science_Normal | Lung_10X_TSP2 | Lung03-NN | Normal | NA | Lung | NA | NA | NA |
| Science_Normal | Mammary_10X_TSP4 | Mam01-NN | Normal | NA | Mammary | NA | NA | NA |
| GSE169246 | GSM5188375 | TNBC01-HM | TNBC | NA | Mammary | NA | NA | rcTxNxM1 |
| GSE169246 | GSM5188399 | TNBC02-HM | TNBC | NA | Mammary | NA | NA | rcTxNxM1 |
| GSE176078 | GSM5354517 | TNBC03-PT | TNBC | III | Mammary | NA | 3 | pT2N0Mx |
| GSE176078 | GSM5354525 | TNBC04-PT | TNBC | III | Mammary | NA | 3 | pT2N1aMx |
| GSE176078 | GSM5354528 | TNBC05-PT | TNBC | III | Mammary | NA | 3 | PT2N0(sn) Mx |
| GSE176078 | GSM5354530 | TNBC06-PT | TNBC | III | Mammary | NA | 3 | pT1cN0 |
| GSE176078 | GSM5354531 | TNBC07-PT | TNBC | III | Mammary | NA | 3 | pT2N1aMx |
| GSE176078 | GSM5354532 | TNBC08-PT | TNBC | III | Mammary | NA | 3 | NA |
| GSE176078 | GSM5354534 | TNBC09-PT | TNBC | III | Mammary | IIA | 3 | pT1cN1Mi |
| Science_Normal | Pancreas_10X_TSP1 | Pan01-NN | Normal | NA | Pancreas | NA | NA | NA |
| Science_Normal | Pancreas_10X_TSP9 | Pan02-NN | Normal | NA | Pancreas | NA | NA | NA |
| GSE197177 | P1_MT | PDAC01-HM | PDAC | NA | Pancreas | NA | NA | NA |
| GSE197177 | P1_T | PDAC01-PT | PDAC | NA | Pancreas | NA | NA | NA |
| GSE197177 | P2_MT | PDAC02-HM | PDAC | NA | Pancreas | NA | NA | NA |
| GSE197177 | P2_N | PDAC02-PN | PDAC | NA | Pancreas | NA | NA | NA |
| GSE197177 | P2_T | PDAC02-PT | PDAC | NA | Pancreas | NA | NA | NA |
| GSE197177 | P3_MT | PDAC03-HM | PDAC | NA | Pancreas | NA | NA | NA |
| GSE197177 | P3_T | PDAC03-PT | PDAC | NA | Pancreas | NA | NA | NA |
| GSE154778 | GSM4679542 | PDAC04-HM | PDAC | IV | Pancreas | IV | NA | NA |
| GSE154778 | GSM4679543 | PDAC05-HM | PDAC | IV | Pancreas | IV | 2 | NA |
| GSE154778 | GSM4679545 | PDAC06-HM | PDAC | IV | Pancreas | IV | 2-3 | NA |
| GSE154778 | GSM4679546 | PDAC07-HM | PDAC | IV | Pancreas | IV | NA | NA |
| GSE154778 | GSM4679547 | PDAC08-HM | PDAC | IV | Pancreas | IV | NA | NA |
| GSE162708 | GSM4957685 | PNET01-HM | PNET | NA | Pancreas | NA | NA | NA |
| GSE162708 | GSM4957683 | PNET01-PT_1 | PNET | NA | Pancreas | NA | NA | NA |
| GSE162708 | GSM4957684 | PNET01-PT_2 | PNET | NA | Pancreas | NA | NA | NA |
| GSE163558 | GSM5004188 | GC01-HM | GC | NA | Stomach | NA | NA | NA |
| GSE163558 | GSM5004180 | GC01-PT | GC | NA | Stomach | NA | NA | NA |
| GSE163558 | GSM5004189 | GC02-HM | GC | NA | Stomach | NA | NA | NA |
| GSE163558 | GSM5004182 | GC02-PT | GC | NA | Stomach | NA | NA | NA |
| OMIX001073 | GC02T | GC03-PT | GC | IIA | Stomach | IIA | T2N1M0 | NA |
| OMIX001073 | GC04T | GC04-PT | GC | IIIB | Stomach | IIIB | T3N3aM0 | NA |
| OMIX001073 | GC05T | GC05-PT | GC | IIA | Stomach | IIA | T1bN2M0 | NA |
| OMIX001073 | GC07T | GC06-PT | GC | IIB | Stomach | IIB | T3N1M0 | NA |
| OMIX001073 | GC08T_S1 | GC07-PT_1 | GC | IA | Stomach | IA | T1bN0M0 | NA |
| OMIX001073 | GC08T_S2 | GC07-PT_2 | GC | IA | Stomach | IA | T1bN0M0 | T1bN0M0 |

*Table S2.*

*Antibodies used for immunofluorescence staining.*

| **Antibody** | **Application** | **Manufacturer** | **Catalog number** | **Dilution** |
| --- | --- | --- | --- | --- |
| CD56 | IF | Proteintech | 60238-1-Ig | 1/500 |
| CD31 | IF | Proteintech | 66065-2-IG | 1/500 |
| CD36 | IF | Proteintech | 66395-1-Ig | 1/500 |
| PANCK | IF | abcam | ab7753 | 1/500 |
| CD68 | IF | Proteintech | 66231-2-IG | 1/500 |
| STMN1 | IF | Proteintech | 82559-1-RR | 1/500 |
| SPP1 | IF | Proteintech | 22952-1-AP | 1/500 |
| CD4 | IF | Proteintech | 67786-1-IG | 1/500 |
| FOXP3 | IF | Proteintech | 22228-1-AP | 1/500 |
| IL-10 | IF | Proteintech | 60269-1-Ig | 1/500 |
