# Supplementary figures and images for: A Single-Cell Atlas of Pan-Cancer Liver Metastasis Reveals Dynamic Cellular Programs Driving Metastatic Progression and Immune Modulation
Source: Research (Wash D C). 2026 Mar 24;9:1208. doi: 10.34133/research.1208 (PMC13010057; doi:10.34133/research.1208)

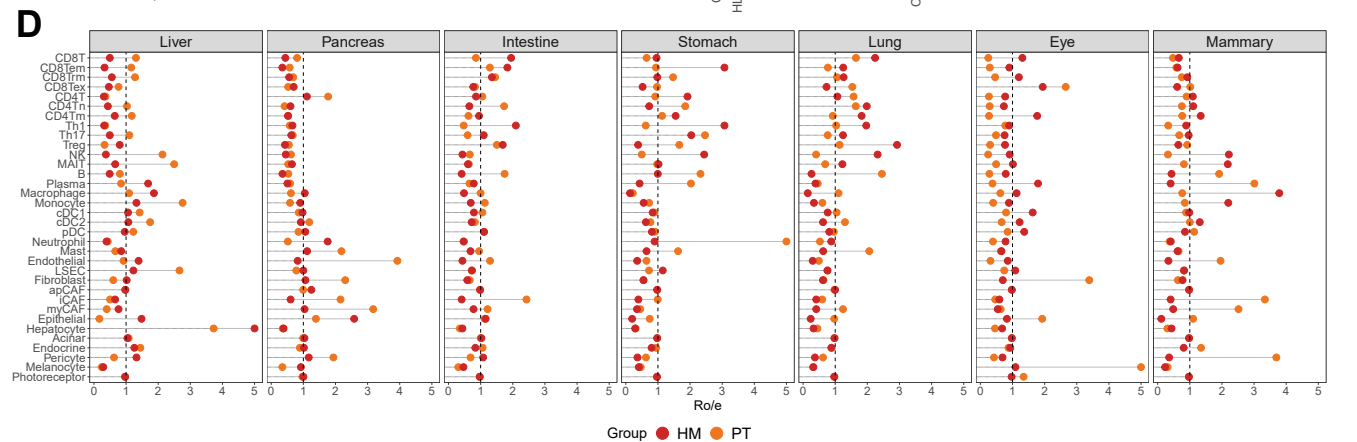

Supplement: Supplementary 1 — Figs. S1 to S7 Tables S1 and S2 [file research.1208.f1.zip › SupplementaryFigure-1.pdf]

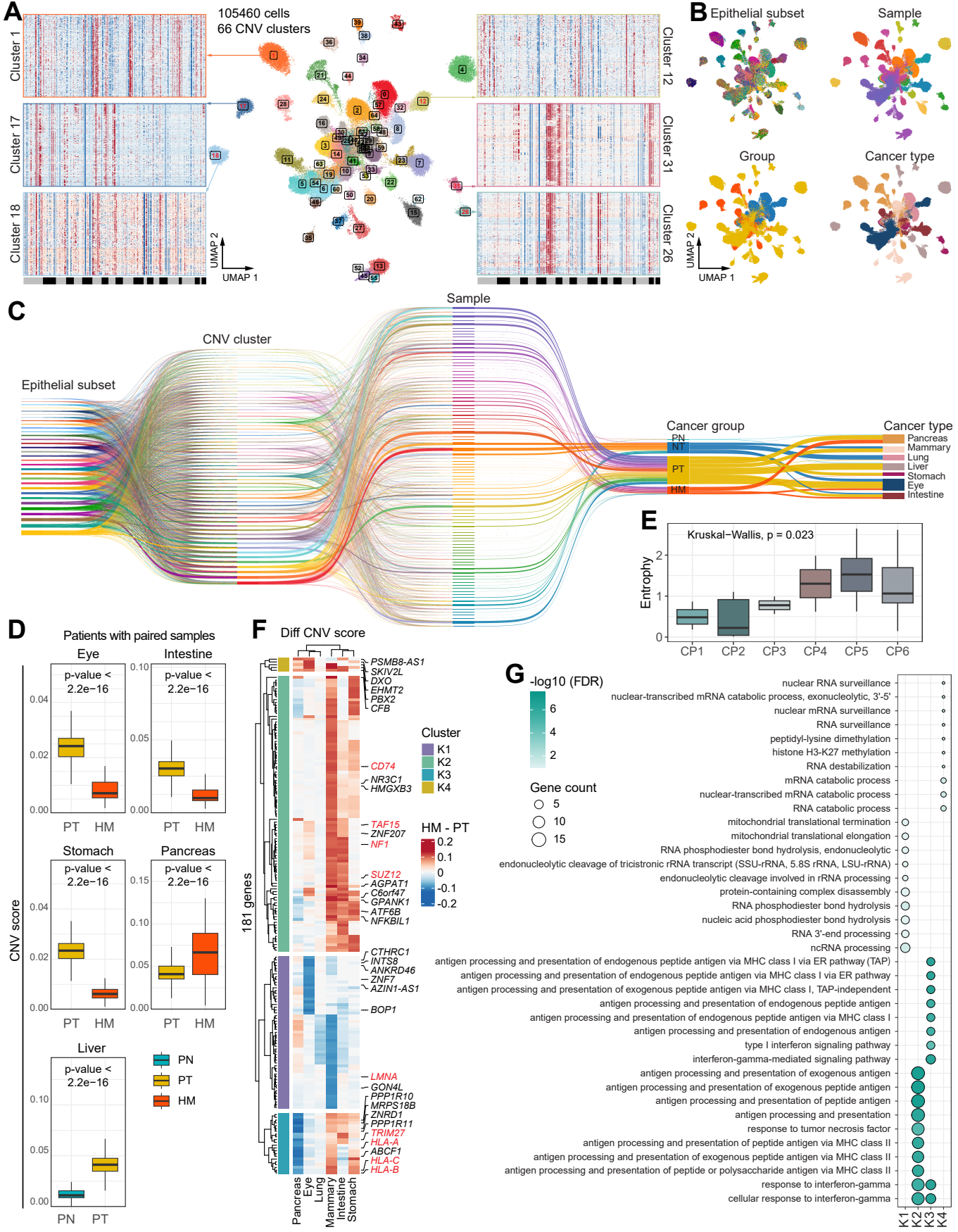

Supplement: Supplementary 1 — Figs. S1 to S7 Tables S1 and S2 [file research.1208.f1.zip › SupplementaryFigure-2.pdf]

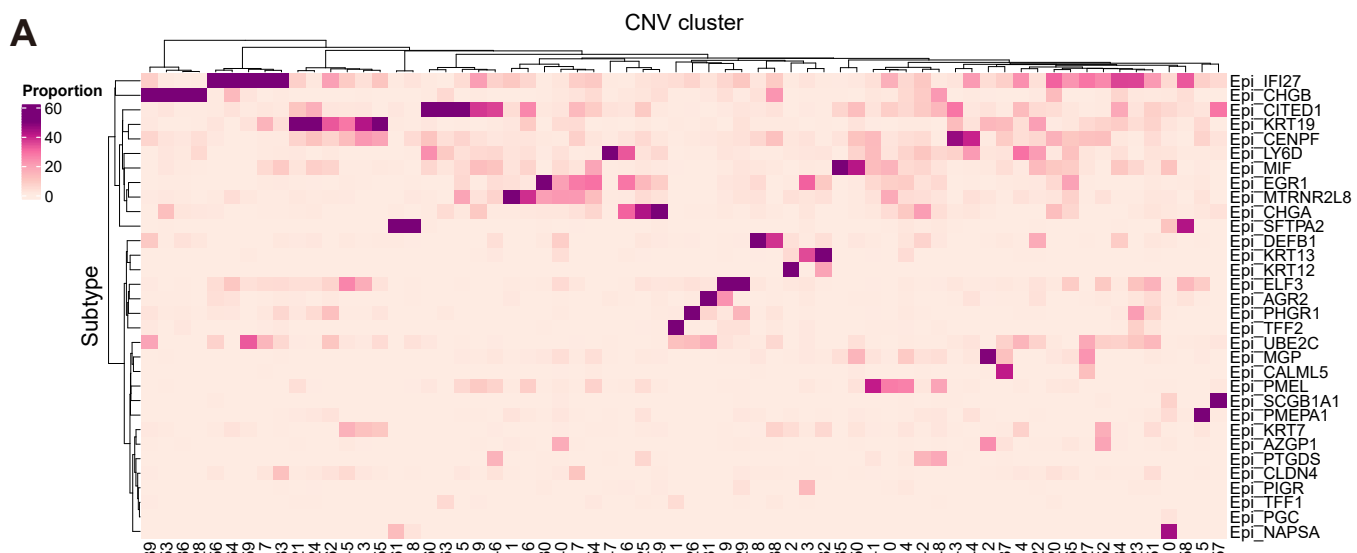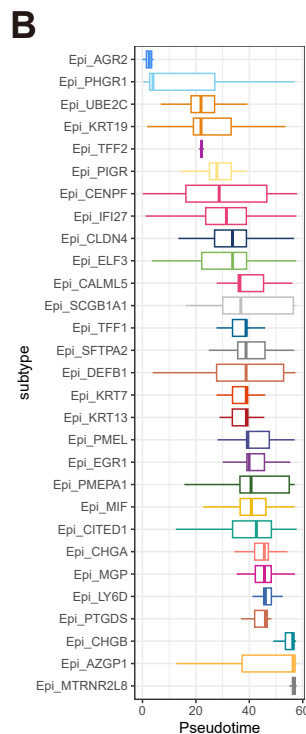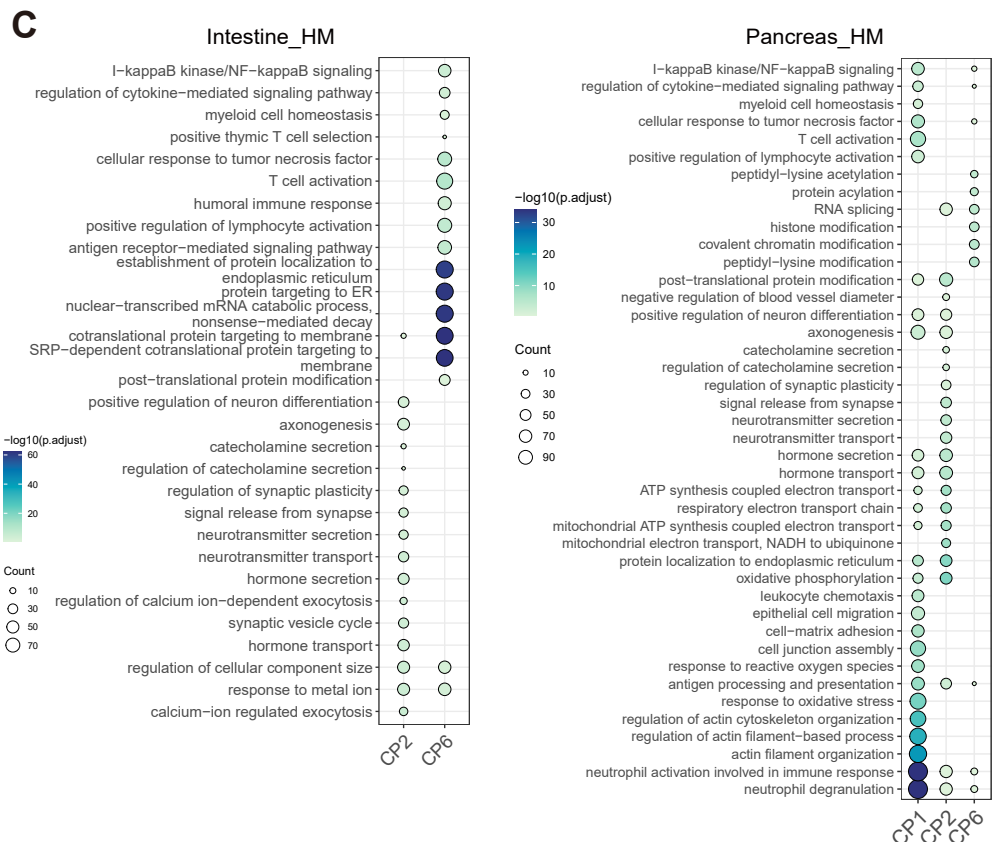

Supplement: Supplementary 1 — Figs. S1 to S7 Tables S1 and S2 [file research.1208.f1.zip › SupplementaryFigure-3.pdf]

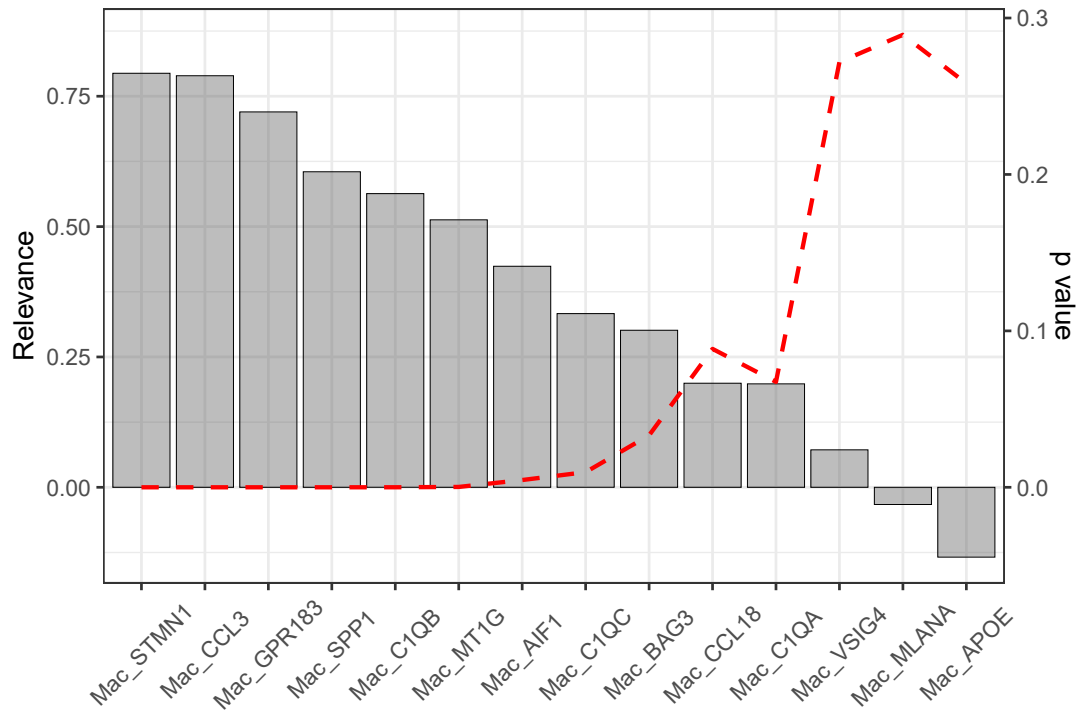

Supplement: Supplementary 1 — Figs. S1 to S7 Tables S1 and S2 [file research.1208.f1.zip › SupplementaryFigure-4.pdf]

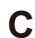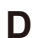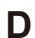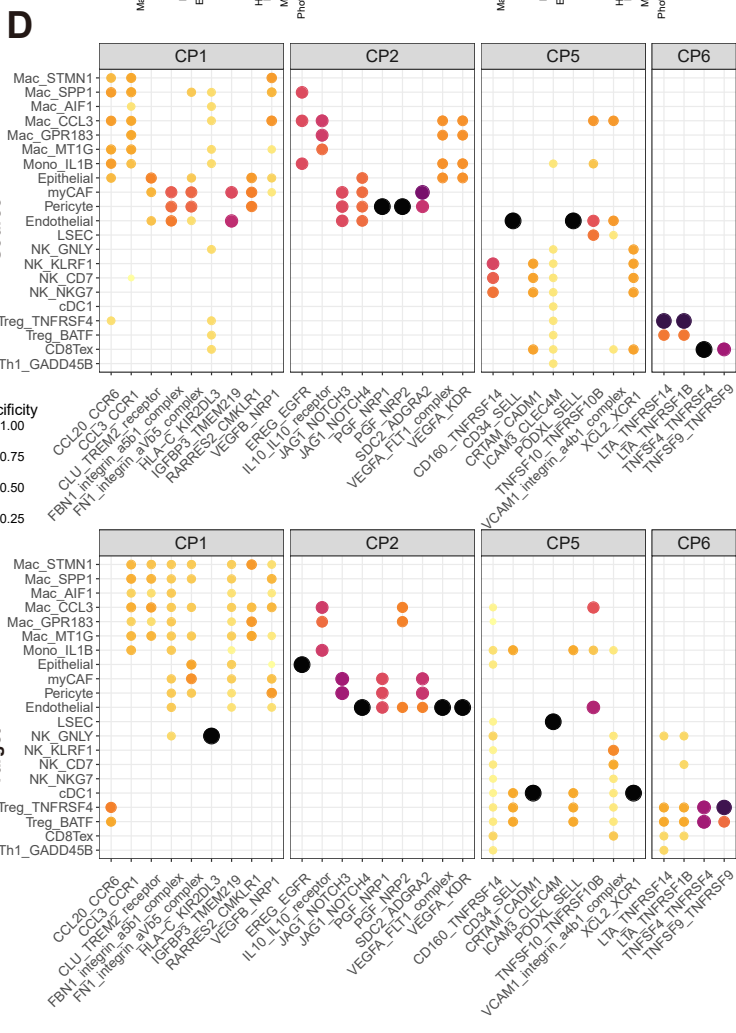

Supplement: Supplementary 1 — Figs. S1 to S7 Tables S1 and S2 [file research.1208.f1.zip › SupplementaryFigure-5.pdf]

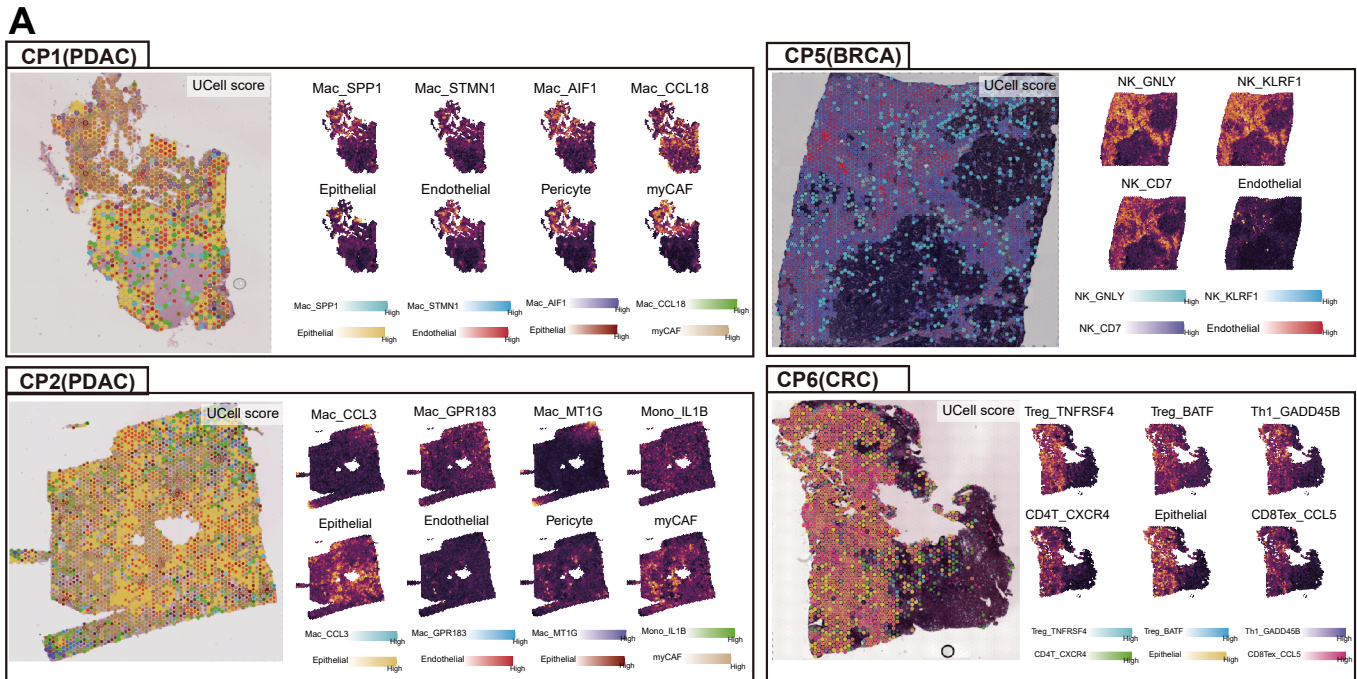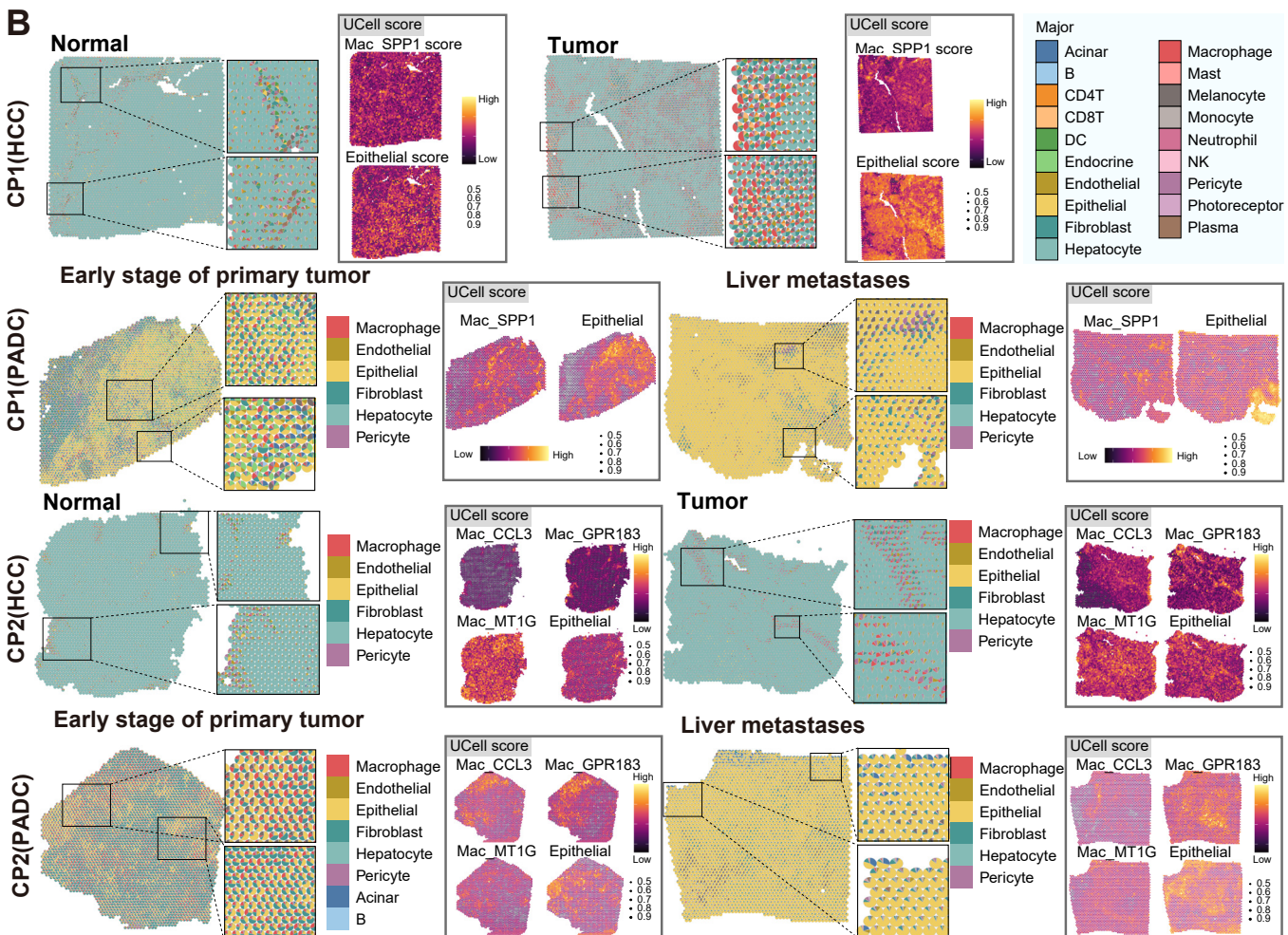

Supplement: Supplementary 1 — Figs. S1 to S7 Tables S1 and S2 [file research.1208.f1.zip › SupplementaryFigure-6.pdf]

# A Early stage of primary tumor

# Liver metastases

B

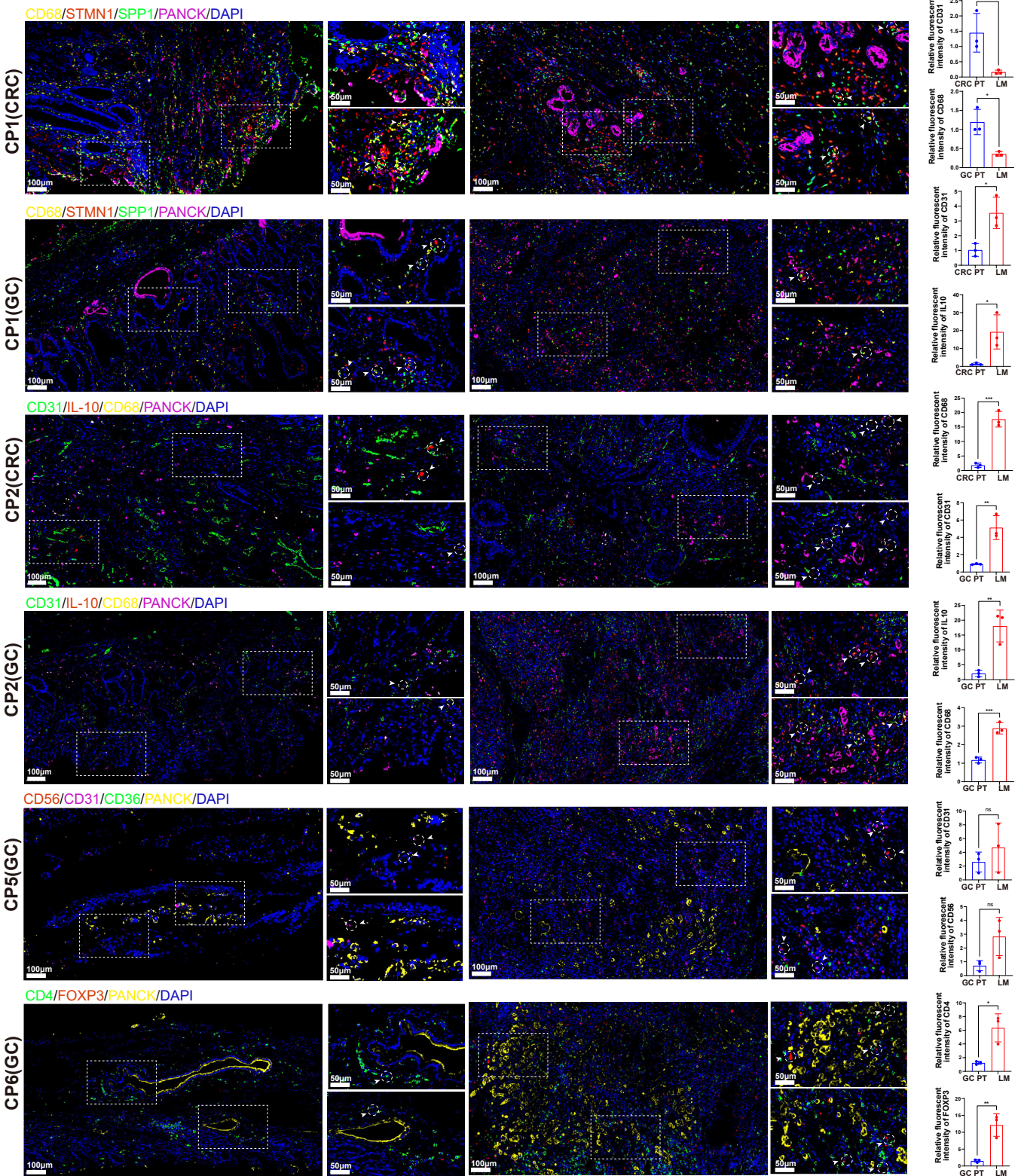

Supplement: Supplementary 1 — Figs. S1 to S7 Tables S1 and S2 [file research.1208.f1.zip › SupplementaryFigure-7.pdf]
